# Assessment of Rice Sheath Blight Resistance Including Associations with Plant Architecture, as Revealed by Genome-Wide Association Studies

**DOI:** 10.1186/s12284-022-00574-4

**Published:** 2022-06-18

**Authors:** Danting Li, Fantao Zhang, Shannon R. M. Pinson, Jeremy D. Edwards, Aaron K. Jackson, Xiuzhong Xia, Georgia C. Eizenga

**Affiliations:** 1 Guangxi Key Laboratory of Rice Genetics and Breeding, Rice Research Institute, Guangxi Academy of Agricultural Sciences, Nanning, Guangxi China; 2 College of Life Sciences, Jiangxi Normal University, Nanchang, Jiangxi China; 3 USDA Dale Bumpers National Rice Research Center, 2890 Highway 130 East, Stuttgart, AR 72160 USA

**Keywords:** Rice, Sheath blight disease, Genome-wide association mapping, Tillering, *Rhizoctonia solani*, *Oryza sativa*

## Abstract

**Background:**

Sheath blight (ShB) disease caused by *Rhizoctonia solani* Kühn, is one of the most economically damaging rice (*Oryza sativa* L.) diseases worldwide. There are no known major resistance genes, leaving only partial resistance from small-effect QTL to deploy for cultivar improvement. Many ShB-QTL are associated with plant architectural traits detrimental to yield, including tall plants, late maturity, or open canopy from few or procumbent tillers, which confound detection of physiological resistance.

**Results:**

To identify QTL for ShB resistance, 417 accessions from the Rice Diversity Panel 1 (RDP1), developed for association mapping studies, were evaluated for ShB resistance, plant height and days to heading in inoculated field plots in Arkansas, USA (AR) and Nanning, China (NC). Inoculated greenhouse-grown plants were used to evaluate ShB using a seedling-stage method to eliminate effects from height or maturity, and tiller (TN) and panicle number (PN) per plant. Potted plants were used to evaluate the RDP1 for TN and PN. Genome-wide association (GWA) mapping with over 3.4 million SNPs identified 21 targeted SNP markers associated with ShB which tagged 18 ShB-QTL not associated with undesirable plant architecture traits. Ten SNPs were associated with ShB among accessions of the *Indica* subspecies, ten among *Japonica* subspecies accessions, and one among all RDP1 accessions. Across the 18 ShB QTL, only *qShB4-1* was not previously reported in biparental mapping studies and *qShB9* was not reported in the GWA ShB studies. All 14 PN QTL overlapped with TN QTL, with 15 total TN QTL identified. Allele effects at the five TN QTL co-located with ShB QTL indicated that increased TN does not inevitably increase disease development; in fact, for four ShB QTL that overlapped TN QTL, the alleles increasing resistance were associated with increased TN and PN, suggesting a desirable coupling of alleles at linked genes.

**Conclusions:**

Nineteen accessions identified as containing the most SNP alleles associated with ShB resistance for each subpopulation were resistant in both AR and NC field trials. Rice breeders can utilize these accessions and SNPs to develop cultivars with enhanced ShB resistance along with increased TN and PN for improved yield potential.

**Supplementary Information:**

The online version contains supplementary material available at 10.1186/s12284-022-00574-4.

## Background

Rice sheath blight (ShB) is a major fungal disease of cultivated rice (*Oryza sativa* L.) worldwide causing yield losses up to 50 percent (Uppala and Zhou 2018). Sheath blight was first reported in Japan in 1910 and subsequently became established in many Asian countries (Lee and Rush 1983; Webster and Gunnell 1992). In China, ShB has become one of the most severe rice diseases (Huang et al. 2009; Zeng et al. 2011). In the southern USA, ShB became prevalent during the 1970s (Webster and Gunnell 1992) with severe yield losses documented in the 1980s (Marchetti and Bollich 1991). Producers apply foliar fungicides to control ShB disease, but tolerant fungal isolates have been discovered (Galam et al. 2021). Only partial resistance or tolerance, henceforth referred to as “resistance”, has been found to date in cultivated rice or wild *Oryza* species gene pools (Li et al. 2021; Molla et al. 2020; Srinivasachary et al. 2011).

The causal agent of sheath blight is the soil-borne, semi-saprophytic fungus *Rhizoctonia solani* Kühn (Webster and Gunnell 1992). *R solani* does not produce spores but rather overwintering sclerotia contact the leaf sheath when rice is flooded and spread up the stem and between tillers and plants using runner hyphae (Uppala and Zhou 2018). The infection then spreads to new plants when these hyphae contact uninfected stems and leaves, resulting in localized areas of infection in the field that are often circular in shape due to the infection spreading radially from an initial point of infection (Srinivasachary et al. 2011). *R. solani* is a necrotrophic fungus, which produces host specific phytotoxins that may act as pathogenicity or virulence factors (Brooks 2007; Vidhyasekaran et al. 1997).

With the goal of identifying genes for ShB resistance that could be introgressed into elite cultivars utilizing marker assisted selection (MAS), a Lemont × TeQing mapping population was evaluated for ShB resistance (Li et al. 1995). Six QTL regions were revealed, but at five of them ShB resistance was associated with undesirable increases in plant height and days to heading, limiting their breeding utility. More than 70 ShB QTL have since been reported across all twelve rice chromosomes using various mapping populations (reviewed by Chen et al. 2019; Jia et al. 2009; Molla et al. 2020; Srinivasachary et al. 2011). Several of these studies also report QTL for plant height and days to heading being co-located with the identified ShB QTL, reinforcing the concern that association with tall plant height and late maturity confounds efforts to incorporate improved ShB tolerance into modern rice cultivars. Of note, Liu et al. (2014) examined additive and epistatic effects, and QTL × environment interactions in the HH1B × RSB02 population and confirmed a negative correlation between ShB severity and plant height. To adjust for these confounding factors, the QTL mapping in the MCR10277 × Cocodrie RIL population using field ShB ratings included plant height and heading as covariates (Nelson et al. 2012). Rosas et al. (2018) similarly used plant height and heading as covariates when genome-wide association (GWA) mapping using field ratings for two other rice sheath fungal diseases, stem rot and aggregate sheath spot. To date, no major large-effect genes for ShB resistance have been found. Only QTL conferring partial resistance have been identified, of which three have been fine-mapped: *qSBR11-1* within the 27.00–28.35 Mb interval on chromosome (chr.) 11 (Channamallikarjuna et al. 2010), *qSB-11*^*LT*^ to the 4.78 to 4.87 Mb region on chr. 11 (Zuo et al. 2013) and *qSB-9*^*TQ*^ to the 21.37 to 21.52 Mb region on chr. 9 (Zuo et al. 2014). Also, Zuo et al. (2014) mapped the *tiller angle control1* (*TAC1*) gene, which gives the TeQing parent a more spreading culm habit, to chr. 9 (20.5–20.9 Mb), close to *qSB-9*^*TQ*^ and noted that the presence of both *qSB-9*^*TQ*^ and *TAC1*^*TQ*^ combined improved ShB resistance. This confirms that an open culm habit affects ShB resistance, possibly by decreasing humidity in the plant canopy. Further evaluation of the advanced generation (F_10:11_) Lemont × TeQing RIL population for ShB resistance brought the number of ShB QTL in that population up to 15 (Pinson et al. 2005) and revealed nine QTL for tiller number per plant (Pinson et al. 2015). Comparing the QTL regions for ShB and TN revealed the two traits mapped to identical QTL regions on chr. 1, chr. 3 and chr. 4. In the HP2216 × Tetep population, ShB and TN QTL overlapped on chr. 3, 5 and 11 (Channamallikarjuna et al. 2010). These studies highlight the fact that for ShB resistance QTL to have breeding value, they must be detected in multiple environments and/or mapping populations and not be associated with yield-reducing plant architecture traits.

To circumvent ShB response data being confounded by these plant architecture traits, six different ShB evaluation methods were developed which involve placing mycelia in agar plugs on plants in the early vegetative stages, or onto detached leaves or tillers, and creating a high humidity environment (Jia et al. 2013; Srinivasachary et al. 2011; Willocquet et al. 2011). The most utilized of these methods is the microchamber method (Jia et al. 2007) which has phenotypically detected segregation of single ShB QTL among near isogenic Lemont × TeQing progeny (Wang et al. 2012), cross-validated ShB QTL based on field evaluations in the Lemont × Jasmine85 RIL population (Liu et al. 2009, 2013) and discovered novel alleles in diverse advance backcross populations involving a wild *Oryza* species donor crossed with a cultivated recurrent parent (Eizenga et al. 2013, 2015, 2022).

Rice has two subspecies, *Indica* (INDAUS) which includes both the i*ndica* (IND) and *aus* (AUS) subpopulations, and *Japonica* (JAP) which includes the *tropical japonica* (TRJ), *temperate japonica* (TEJ) and aromatic (ARO) subpopulations (Cheng et al. 1984; Garris et al. 2005; Kato et al. 1930). To better understand the genetic basis of complex traits, GWA mapping panels like the Rice Diversity Panel 1 (RDP1) and USDA-ARS Rice Minicore (RMC) were developed. The RDP1 includes 424 *O. sativa* accessions which represent the world-wide diversity in rice with accessions from the five major subpopulations (Eizenga et al. 2014; Zhou et al. 2016). Initially RDP1 was genotyped with 44,000 SNP markers, which was expanded to 700,000 SNPs with the High-Density Rice Array (McCouch et al. 2016) and recently expanded to 4,829,392 SNPs by imputation (Wang et al. 2018). The RMC consists of 217 *Oryza* accessions and represents the diversity in the USDA-ARS rice germplasm collection (Agrama et al. 2009). The 202 *O. sativa* accessions in the RMC represent the five major subpopulations and the entire RMC was genotyped with 155 simple sequence repeat (SSR) markers (Jia et al. 2012). More recently, 173 RMC *O. sativa* accessions were genotyped with 3.2 million SNPs (Huggins et al. 2019; Wang et al. 2016).  To identify putative alleles for ShB resistance, the entire RMC was phenotyped for ShB using the microchamber method and alleles based on ten different SSR markers distributed across seven chromosomes were significantly associated with ShB resistance (Jia et al. 2012). With the high-density genotypes now available for both the RDP1 and RMC, it is possible to unravel the complex interactions and putative genes underlying the partial resistance to *R. solani* exhibited by some rice accessions.

The main objective of this study was to identify rice accessions in the RDP1 carrying novel alleles for ShB resistance to deploy against virulent *R. solani* isolates prevalent in Arkansas, USA (AR) and Nanning, China (NC). To this end, the RDP1 was evaluated for ShB resistance in both AR and NC using one endemic *R. solani* isolate, at the seedling stage in the greenhouse to alleviate the confounding effects of plant architecture (days to heading, plant height, culm habit, tillering). Field studies were conducted to further confirm the ShB resistance, using days to heading and plant height as covariates, as previously mentioned, to identify ShB QTL not confounded by these traits. Increased tillering and panicle production were evaluated in a greenhouse study. GWA studies were conducted for all the aforementioned traits to determine if the associated marker-trait QTL mapped to the same regions, thus confirming ShB QTL and revealing those that were not confounded by undesirable plant architecture traits. To provide additional confirmation of ShB-QTL, the ShB GWA mapping for the RMC was reanalyzed with the recently available SNP genotypes.

## Results

### Disease Reactions Stronger in Nanning, China than Arkansas, USA

**Fig. 1:**
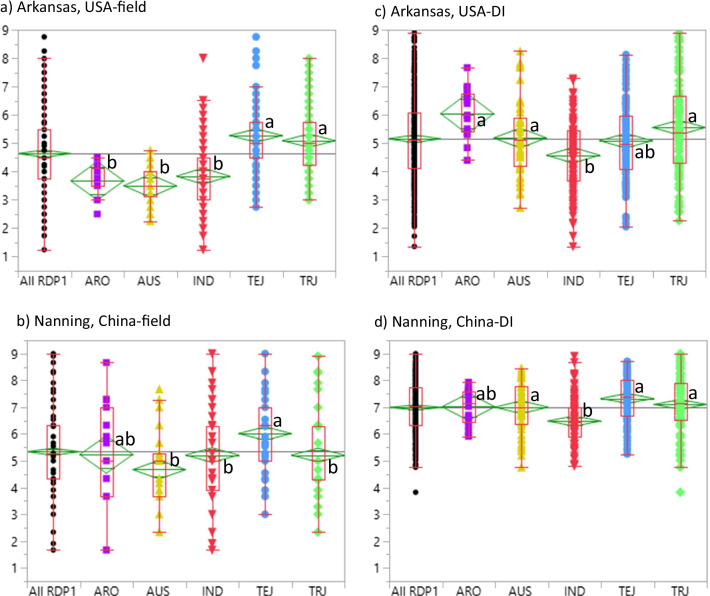
Quantile plots comparing sheath blight disease ratings (0 = no disease to 9 = 90% or more infected) between the Rice Diversity Panel 1 (All-RDP1) and the *O. sativa* subpopulations, *aromatic* (ARO), *aus* (AUS), *indica* (IND), *temperate japonica* (TEJ) and *tropical japonica* (TRJ). The ratings were from the field (**a**, **b**) and microchamber disease index (DI) (**c**, **d**) studies conducted in Arkansas, USA (**a**, **c**) and Nanning, China (**b**, **d**). Dots indicate full ranges of observed data; green diamonds indicate means ± se; lower and upper sides of red quantile boxes indicate the 25th and 75th percentiles, respectively; horizontal red lines in boxes are medians, vertical lines indicate the 5th and 95th percentiles. Different lowercase letters indicate differences between subpopulation means for that trait based on Tukey-Kramer multiple mean comparison tests and α = 0.05

ShB severity was evaluated in replicated inoculated field studies over two years in AR during 2016 (AR16), 2017 (AR17), as a two year mean in AR (ARall, in 2018 in NC (NC18), and using a microchamber disease index (DI) in both AR (AR-DI) and NC (NC-DI). Comparing both field and microchamber evaluations, ShB disease was more severe in NC than in AR and mean comparisons across the five subpopulations generally ranked TEJ with the higher mean rating or more susceptible, while IND had lower mean ratings, thus more resistant (Fig. 1, Table 1). Mean comparisons of AR and NC field ratings clustered AUS with the lower ShB ratings among subpopulations but clustered the TRJ with the subpopulations having higher ratings in AR fields and lower ratings in NC fields. This difference is particularly noteworthy because the disease severity was higher in NC than in AR.

**Table 1:** Means and standard deviations for each trait, across the All RDP1 and each *O. sativa* subpopulation: *aus* (AUS), *indica* (IND*), temperate japonica* (TEJ), *tropical japonica* (TRJ), aromatic (ARO) and admixtures of subpopulations (Admix)

Trait—study	RDP1 (n = 424)	AUS (n = 62)	IND (n = 102)	TEJ (n = 116)	TRJ (n = 113)	ARO (n = 15)	Admix (n = 16)
	Avg ± stdev	Avg ± stdev	Avg ± stdev	Avg ± stdev	Avg ± stdev	Avg ± stdev	Avg ± stdev
ShB-field ARall (2 years)	4.65 ± 1.33	3.50 ± 0.62b	3.84 ± 1.33b	5.27 ± 1.15a	5.09 ± 1.16a	3.67 ± 0.56b	4.31 ± 1.13ab
ShB-field NC18 (1 year)	5.35 ± 1.41	4.67 ± 1.13b	5.21 ± 1.48b	6.02 ± 1.21a	5.21 ± 1.38b	5.23 ± 1.83ab	4.90 ± 1.24b
ShB-DI AR	5.17 ± 1.43	5.19 ± 1.26ab	4.58 ± 1.24b	5.09 ± 1.33ab	5.56 ± 1.62a	6.04 ± 0.90a	5.83 ± 1.39a
ShB-DI NC	7.01 ± 0.97	7.00 ± 0.96a	6.49 ± 0.89b	7.33 ± 0.85a	7.11 ± 1.04a	7.02 ± 0.66ab	7.32 ± 0.83a
Plant height-ARall (2 years)	136.5 ± 21.8	154.4 ± 8.4a	139.5 ± 24.3b	123.6 ± 16.2c	138.2 ± 20.6b	165.3 ± 12.0a	136.5 ± 20.2b
Plant height-NC18	129.5 ± 21.0	141.8 ± 14.3a	132.0 ± 21.9b	116.3 ± 17.7c	132.5 ± 19.2b	143.9 ± 23.0a	130.8 ± 19.9b
Days to heading-ARall (2 years)	85.3 ± 13.0	82.3 ± 8.0b	87.2 ± 14.3ab	82.5 ± 13.4b	87.1 ± 11.7ab	94.5 ± 16.8a	83.1 ± 13.2b
Days to heading-NC18	66.3 ± 6.7	66.1 ± 3.6ab	67.9 ± 6.1ab	61.3 ± 6.3b	69.9 ± 6.0a	70.8 ± 4.0a	65.1 ± 6.8ab
Culm habit-AR17	1.96 ± 0.79	2.76 ± 0.64a	2.38 ± 0.81b	1.61 ± 0.60c	1.71 ± 0.66c	2.50 ± 0.58a	2.08 ± 0.79b
Tiller no. at 5–6 weeks	4.09 ± 1.04	4.10 ± 1.28b	4.80 ± 1.07a	4.00 ± 0.78b	3.56 ± 0.76c	3.74 ± 0.78b	4.15 ± 1.09b
Panicle no. at maturity	3.30 ± 0.87	3.71 ± 0.94a	3.77 ± 0.72a	3.22 ± 0.80b	2.73 ± 0.67c	3.45 ± 0.90a	3.22 ± 0.63b

Lowercase letters indicate differences among subpopulation means per trait at the α = 0.05 level, determined using the Tukey–Kramer multiple comparison tests

After classifying the accessions per study as resistant (R), moderately resistant (MR), moderately susceptible (MS), or susceptible (S) (details in Materials and Methods), the number and percentage of each subpopulation in each disease response category were determined (Table 1). In accordance with the differences observed among subpopulation means, the AR-field and AR–DI data identified more accessions as R than did the NC datasets, and IND had a higher number and proportion of R accessions than the other subpopulations. Even though disease severity was stronger in NC fields than in AR fields, more TRJ accessions were R in NC (6 accessions, 5.5%) than in AR (1, 0.9%). In contrast, the AR-field data identified 25 IND (34.25%) as R, while NC-field data identified only 8 (8.25%). NC-DI disease levels were so severe that no accessions were categorized as R (Table 1, Fig. 1). Another noticeable difference between field and microchamber data is that in both AR and NC, the AUS and ARO accessions appeared more R in field plots than in microchamber evaluations (Table 1).

### Correlations Between Sheath Blight Ratings Across Locations and Evaluation Methods

Field ratings were better correlated between the two AR years (r = 0.55) than between the two geographically diverse locations, AR and NC (r = 0.30, Table 2). Data from microchamber evaluations across the two locations had a similarly low correlation coefficient (r = 0.31, Table 2). Each country used a different, local *R. solani* isolate in their ShB evaluations, and differences in isolate virulence (Wamishe et al. 2007) might be contributing to the low correlations observed between AR and NC field and DI ratings. If the low AR-NC correlations seen for field data are being caused by differences in host resistance to the NC and AR isolates, then it would logically follow that the DI data would better predict field responses in their respective country than across countries. What was observed, however, was stronger correlation between AR-field ratings with NC-DI (r = 0.40, Table 2) than with AR-DI (r = 0.21), and NC-field ratings were poorly predicted by either NC-DI or AR-DI (both r < 0.1). The Chinese *R. solani* isolate was in fact as good as the US isolate at predicting field R. Furthermore, the AR-field ratings were a better predictor of NC-field responses (r = 0.30, Table 2) than were either AR-DI or NC-DI.

**Table 2:** Pearson correlations among traits

ALL RDP1 correlations	ShB-field NC	ShB-DI AR	ShB-DI NC	Plant height—AR	Plant height—NC	Days to heading—AR	Days to heading—NC	Culm habit	Tiller number	Panicle number
ShB-field AR	0.30***	0.21***	0.40***	− 0.66***	− 0.41***	− 0.17**	− 0.23***	− 0.40***	− 0.21***	− 0.19***
ShB-field NC		0.09	0.09	− 0.39***	− 0.65***	− 0.08	− 0.41***	− 0.11	− 0.07	− 0.10
ShB-DI AR			0.31***	− 0.05	− 0.07	− 0.09	− 0.01	− 0.06	− 0.21***	− 0.19***
ShB-DI NC				− 0.23***	− 0.16**	− 0.19***	− 0.11	− 0.21***	− 0.24***	− 0.15**
Plant height—AR					0.66***	0.20***	0.33***	0.43***	− 0.07	− 0.02
Plant height—NC						0.16**	0.42***	0.28***	− 0.005	0.08
Days to heading—AR							0.27***	0.01	0.04	0.05
Days to heading—NC								−0.002	0.07	−0.04
Culm habit									0.18***	0.21***
Tiller number (TN)										0.68***

Sheath blight (ShB) response was evaluated in Arkansas, USA (AR) and Nanning, China (NC) using both field scoring and microchamber disease index (DI). Correlation values (r) are bold if significantly positive, italics if significantly negative, and black if not significant at α = 0.05. Significance at α = 0.01 and α = 0.001  are indicated with ** and ***, respectively

Analyses of the data collected at NC and observations by those conducting the study (D Li and X Xia, personal communication, 2019) did not reveal what factor(s) caused the NC-field data to be so unique that it correlated poorly with the other disease datasets. No other disease pressure or unusual circumstances were reported at the NC field study; thus, these differences are noted but unexplained.

### Field-Sheath Blight and Plant Height were Strongly Negatively Correlated Across Subpopulations and Locations

Among the strongest of all trait-to-trait correlations observed were those between ShB-field and plant height (Table 2). The three subpopulations with tallest mature plant height, namely ARO, AUS, and IND (Table 1), also exhibited proportionally more field ShB resistance among their accessions than the other subpopulations (Additional file 1: Table S1), raising concern that the comparatively tall height of the R accessions in these three subpopulations might be creating or biasing the strong negative correlations seen between field resistance and tall plant height in the RDP1 (r = − 0.62 AR, r = − 0.53 NC, Table 2)**,** and causing large portions of the field disease variance to height differences in both AR (*R*^2^ = 38%) and NC (*R*^2^ = 28%). We therefore also examined correlations between field-ShB and plant height within each subpopulation and found them to be relatively strong and negative within the IND, TEJ, and TRJ subpopulations in both AR and NC (Additional file 1: Table S2), and in the ARO and AUS NC data, but insignificant for the ARO and AUS in AR. The ARO and AUS subpopulations had smaller sample sizes in AR (n = 13 and 37, respectively) than in NC (n = 15 and 67 respectively), since the accessions with red pericarp could not be grown in AR fields. Height observed in the two countries correlated well (r = 0.66, Table 2), and the ranking of the subpopulations for average height was identical in both countries (Table 1).

### Correlations Among Additional Plant Traits and Associations with Sheath Blight Severity

On average accessions flowered 19 days earlier in NC than in AR fields, likely impacted by photoperiod differences. Positive correlations between plant height and days to heading were seen in both locations (Table 2) and across each of the individual subpopulations (Additional file 1: Table S2), with taller plants generally having longer vegetative growth periods. While widely spreading culms are undesirable because they impede harvest, whether by machine or hand, they showed desirable association with reduced ShB severity, likely due to increased airflow through the more open plant canopies.

Correlations between TN and PN were among the strongest trait-to-trait correlations observed in this study (r = 0.68, Table 2). The TN-PN correlation was weaker in the TRJ subpopulation (r = 0.61) than the others and was strongest among the AUS (r = 0.80) (Additional file 1: Table S2). Among the subpopulations, TRJ is also notably low for both TN and PN (Table 1), and, except for the ARO which had few accessions (n = 15), the TRJ also had the narrowest range for TN, while the AUS exhibited the highest variance and standard deviation (variance = stdev^2^) for TN (Table 1). Increased TN and decreased culm habit (more erect tillers) can each restrict airflow through the plant canopy. Such reduced airflow is consistent with the negative correlation observed between culm habit and field ShB (Table 2), but not supported by the negative correlation between TN and field ShB (− 0.21, α = 0.0002) which suggests increased TN does not increase ShB severity but is instead associated in some way with decreased ShB.

### Sheath Blight QTL Identified from GWA Studies Across Subpopulations

**Fig. 2:**
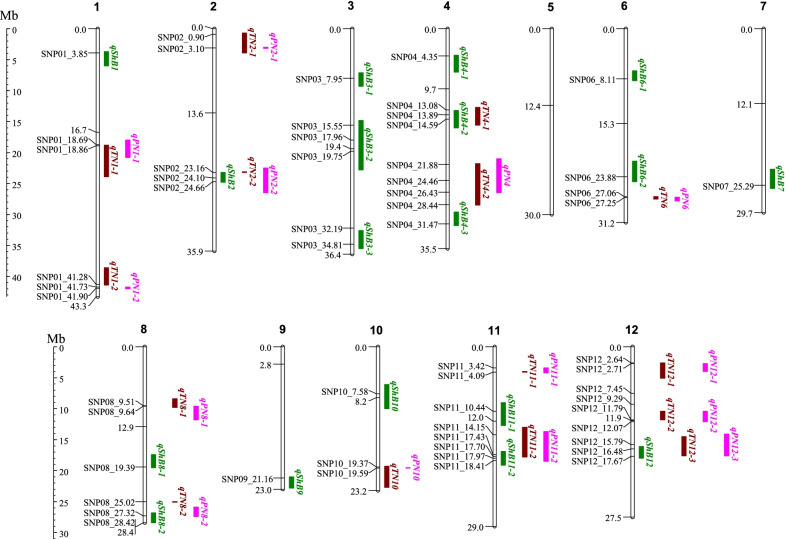
The physical position of the sheath blight QTL (*qShB*), panicle number QTL (*qPN*) and tiller number QTL (*qTN*) identified by genome-wide association (GWA) mapping in the Rice Diversity Panel-1 (RDP1) with 3,463,224 SNP markers across the entire rice genome. The 21 SNPs ascertaining the 18 *ShB-*QTL, 18 SNPs defining the 14 *PN*-QTL and 19 SNPs delineating the 15 *TN*-QTL are identified by “SNP”, chromosome and megabase (Mb) position based on the Os-Nipponbare-Reference-IRGSP-1.0 assembly (Kawahara et al. 2013). The Mb position of the centromere, and the beginning and end of each chromosome, is given. The details of the ShB-QTL and distinguishing SNPs are in Table 3, *PN*-QTL and *TN*-QTL in Table 4, and the QTL are combined in Table S5. [The figure was created with MapChart 2.32 (Voorrips 2002).]

From the GWA analyses of ShB ratings in the field and DI in the greenhouse, 330 significant marker-trait associations were found across all seven panels (395, INDAUS, JAP, IND, AUS, TRJ, TEJ) which tagged 309 of the over 3.4 million SNPs included in the analyses (Additional file 1: Table S3). Overall, 210 marker-trait associations were with field ratings, 120 with DI, 259 associations were identified from ShB ratings collected in AR and 71 from ratings in NC. From these analyses, 18 ShB GWA QTL regions (Table 3, Fig. 2) were ascertained based on 145 marker-trait associations from 135 unique SNPs (Additional file 1: Table S4). Selection of these 18 ShB QTL regions was based on the region having three or more associated SNPs in close proximity, using both the Manhattan plots (Additional file 2: Fig. S1) to determine if there were supporting significant SNPs even though some were below the threshold (thus not in Additional file 1: Table S3) and Q-Q plots to compare the distribution of observed and expected *p*-values (Additional file 2: Fig. S1). Additional factors in selecting ShB QTL regions include the allele frequency of the peak SNP and consistent associations with both field and greenhouse ratings across locations and the different panels. The relationship between each SNP allele and trait means within subpopulations were subsequently examined using pivot tables, resulting in 21 significantly associated SNPs being selected as “targeted SNPs” to highlight the 18 ShB QTL regions (Fig. 2, Table 3, Additional file 1: Table S4).

**Table 3:** QTL associated with sheath blight (ShB) tolerance in the Rice Diversity Panel 1 (RDP1), arranged in chromosomal order to indicate when a QTL region associated with more than one trait

QTL	Chr	Start of QTL region (bp)	End of QTL region (bp)	QTL size (bp)	Peak SNP (bp)^a^	-log_10_(*p*)	Trait^b^	Study location, year^c^	Panel^d^	Refer-ence allele^e^	Reference allele effect^e^	Candidate gene(s)		
												MSU ID^f^	Gene symbol(s)^g^ or name(s)	Citation
*qShB1*	1	3650475	6013771	2363296	3852467	5.35	ShB-field	AR17	AUS	C	− 1.03	LOC_Os01g08710	*OsWRKY102*	Pooja et al. (2015)
*qShB2*	2	23195252	24805469	1610217	24095212	5.58	ShB-DI	NC-gh	INDAUS	C	0.51	LOC_Os02g39330	*CHITINASE 6*	Nakazaki et al. (2006)
*qShB3-1*	3	7054964	9262436	2207472	7210477	5.83	ShB-field	AR17	TRJ	T	− 0.92	LOC_Os03g13210	*POXN*	Gupta et al. (2012)
*↓*												LOC_Os03g13820	*OsRLCK105*	Vij et al. (2008)
*↓*												LOC_Os03g14000	*OsACBP5*	Liao et al. (2020), Panthapulakkal Narayanan et al. (2019)
*qShB3-2*	3	14819510	22780248	7960738	15552510	5.58	ShB-DI	AR-gh	JAP	T	0.77	LOC_Os03g27120	*OsMC1*	Wang and Zhang (2014), Huang et al. (2015)
*↓*					17960988	9.57	ShB-DI	AR-gh	TRJ	A	− 0.94	LOC_Os03g27170	*OsMC3*	Wang and Zhang (2014), Huang et al. (2015)
*↓*					19749554	6.12	ShB-field	AR16	TEJ	C	0.86	LOC_Os03g27190	*OsMC8*	Wang and Zhang (2014), Huang et al. (2015)
*↓*												LOC_Os03g27210	*OsMC2*	Wang and Zhang (2014), Huang et al. (2015)
*↓*												LOC_Os03g28940	TIFY10A	Shimizu et al. (2013)
*↓*												LOC_Os03g32314	*OsAOC*	Yoeun et al. (2018), Yu et al. (2020)
*↓*												LOC_Os03g33012	*OsWRKY33*	Shi et al. (2020)
*qShB3-3*	3	31569875	35543780	3973905	32191693	5.20	ShB-field	ARall	INDAUS	C	1.16	LOC_Os03g56820	*FAH2*	Nagano et al. (2016)
*↓*					34808860	5.41	ShB-field	ARall	AUS	A	− 0.71	LOC_Os03g60710	*OsRLCK118*	Li et al. (2017)
*qShB4-1*	4	4302304	7036317	2734013	4352304	5.21	ShB-field	ARall	AUS	G	− 0.72	LOC_Os04g10060	*OsKS4*	Park et al. (2012)
*qShB4-2*	4	13238768	16035378	2796610	13889641	5.75	ShB-field	NC18	395	T	0.46	LOC_Os04g24220	*OsWAK32*	Yuan et al. (2018)
*↓*												LOC_Os04g24510	*OsWAK36*	Yuan et al. (2018), Zhang et al. (2005)
*↓*												LOC_Os04g27860	*OsRPS27*	Saha et al. (2017)
*qShB4-3*	4	29559997	31797355	1961020	31471017	6.23	ShB-field	AR17	TRJ	G	− 0.94	LOC_Os04g51460	*OsXET9*	Dong et al. (2011)
*qShB6-1*	6	6804694	8440362	1635668	8112438	6.37	ShB-field	ARall	JAP	T	− 0.48	LOC_Os06g14540	GH9B3	Huang et al. (2019)
*qShB6-2*	6	21393533	24656415	3262882	23884567	5.67	ShB-DI	AR-gh	TRJ	C	− 0.67	LOC_Os06g40790	*OsMLO7*	Nguyen et al. (2016)
*↓*												LOC_Os06g41010	*OsSAP1*	de Freitas et al. (2019)
*qShB7*	7	22723331	25791908	3068577	25286267	5.54	ShB-field	ARall	TRJ	G	− 0.83	LOC_Os07g42370	TIFY10B	Ye et al. (2009)
*qShB8-1*	8	17404835	19452954	2048119	19393988	6.02	ShB-DI	NC-gh	AUS	A	0.96	LOC_Os08g29660	*OsWRKY69*	Berri et al. (2009), Choi et al. (2017)
*qShB8-2*	8	26787346	28440581	1653235	28417969	7.41	ShB-DI	NC-gh	AUS	A	− 0.81	LOC_Os08g42580	*OsCERK1*	Carotenuto et al. (2017)
*↓*												LOC_Os08g42700	PIB*H8*	Wang et al. (2001)
*↓*												LOC_Os08g44400	*OsGRX20*	Ning et al. (2018)
*↓*												LOC_Os08g44480	*OsRPS25*	Saha et al. (2017)
*qShB9*	9	21000812	22806305	1805493	21159446	5.96	ShB-DI	NC-gh	INDAUS	C	− 0.42	LOC_Os09g37006	*OsAlba7*	Verma et al. (2018)
*↓*												LOC_Os09g37270	*OsRacGEF2*	Akamatsu et al. (2015)
*↓*												LOC_Os09g39910	*OsABC9*	Oh et al. (2021)
*qShB10*	10	6072028	10023439	3951411	7584959	6.28	ShB-field	AR17	TRJ	C	− 0.92	LOC_Os10g11500	PR1-101	Mitsuhara et al. (2008)
*↓*												LOC_Os10g11980	*OsAT1*	Mori et al. (2007)
*↓*												LOC_Os10g13550	IREN	Ootsubo et al. (2016)
*qShB11-1*	11	8996571	12711977	3715406	10435614	5.36	ShB-DI	AR-gh	JAP	C	− 0.85	LOC_Os11g17380	*OsRLCK321*	Vij et al. (2008)
*qShB11-2*	11	16919914	19065774	2145860	17974233	5.39	ShB-DI	NC-gh	IND	C	1.03	LOC_Os11g31190	SWEET14	Blanvillain-Baufume et al. (2017)
*qShB12*	12	16108674	17979174	1870500	17674629	6.40	ShB-DI	AR-gh	AUS	G	− 0.95	LOC_Os12g29220	SWEET13	Liu et al. (2011)
*↓*												LOC_Os12g29430	*OsWAK125*	Dixit et al. (2015), Zhang et al. (2005)
*↓*												LOC_Os12g32820	*OsABC12*	Oh et al. (2021)

^a^*O. sativa* SNPs are identified by their physical location based on the Os-Nipponbare-Reference-IRGSP-1.0 assembly (Kawahara et al. 2013)

^b^Sheath blight severity was rated (0 = resistant, 9 = severe) using both field (ShB-field) and microchamber (ShB-DI) studies. The field studies in Arkansas had two replications each year and the field study in China and the microchamber studies each had three replications

^c^AR16 and AR17 indicates GWA-mapping used trait BLUPs calculated across two replications evaluated in Arkansas, USA in 2016 and 2017, respectively, while ARall indicates BLUPs calculated across both years, and NC18 indicates BLUP data were from the single-year study with three replications conducted in Nanning, China in 2018

^d^Panels are defined as the complete Rice Diversity Panel 1 (395) and subpopulation groups*, tropical japonica* (TRJ), *temperate japonica* (TEJ), *au*s (AUS) and *indica* (IND). The two *O. sativa* subspecies *Indica* (INDAUS) comprised of IND and AUS and *Japonica* (JAP) comprised of TEJ and TRJ. QTL were often identified in GWA-mapping of more than one population (e.g., in INDAUS and AUS); the table presents the results based on the least-complex population in which the QTL was found significant

^e^Reference allele based on the Os-Nipponbare-Reference-IRGSP-1.0 assembly (Kawahara et al. 2013). A negative allele effect at a ShB locus reflects reduced disease severity, or increased tolerance

^f^MSU ID is the Rice Genome Annotation Project locus identified for the candidate gene (Kawahara et al. 2013)

^g^Gene nomenclature followed the standardized nomenclature for rice genes used in *Oryzabase* (Yamazaki et al. 2010)

Plant height and heading were included as covariates in the GWA analyses of field ShB ratings in order to control for their effect on field ShB ratings and increase the likelihood of identifying ShB QTL due to physiological or biochemical resistance mechanisms. To verify the ShB QTL were independent from height and maturity, the 18 ShB QTL regions were examined for SNPs significantly associated with height and heading identified by the RDP1 (395) GWA mapping (Additional file 1: Table S3, Additional file 2: Fig. S2). Only *qShB6-1* encompassed more than one SNP significantly associated with height or heading. In fact, there were two SNPs associated with heading within *qShB6-1* and six additional SNPs associated with heading within 1 Mb of this QTL. For the *qShB6-1* targeted SNP, SNP06_8.112438 (Additional file 1: Table S4), the relationship between the alleles and mean days to heading was examined using pivot tables, revealing that the T allele associated with ShB resistance in JAP was associated with increased days to heading in the TEJ but decreased days to heading in TRJ with a range of one to nine days. Of note, the *PHOTOSENSITIVITY 1* (*Hd1*) gene which promotes flowering under short days (Wei et al. 2016) is at 9.34 Mb, near *qShB6-1,* and may be influencing this days to heading QTL.

Culm habit, whether a plant is upright or has a more open canopy, is the third trait that can confound field ShB rating. GWA mapping for culm habit in RDP1 (395), identified a single SNP on chr. 9, SNP09_3.441587, significantly associated with culm habit, which was not near any ShB GWA-QTL we report (Additional file 1: Table S3).

Across the 21 targeted marker-ShB associations tagging the 18 ShB-QTL (Table 3), 11 corresponded to field ratings with 10 from ratings in AR and one from ratings in NC, and 10 associations corresponded with DI of which five were conducted in AR and five in NC (Table 3). Across the seven panels, six SNPs each were associated with ShB in AUS and TRJ; three SNPs each with INDAUS and JAP; and one SNP each with RDP1, IND and TEJ.

Of note, three of the four longest ShB QTL regions, *qShB3-2* (7.96 Mb), *qShB10* (3.95 Mb) and *qShB11-1* (3.72 Mb), all included the centromeric region (Fig. 2, Table 3). These longer QTL regions may be due to lower recombination in the centromeric region.

To evaluate the ability to use the SNPs identified here as tagging ShB QTL within the RDP1 population to predict the presence and frequency of the R allele in accessions outside of RDP1, we compared the RDP1 allele frequencies at the targeted SNPs (Additional file 1: Table S5) with those reported in a larger collection (4726 accessions) using RiceVarMap2 (Zhao et al. 2015) or Rice SNP-Seek (3000 accessions; Mansueto et al. 2017) for SNP01_3,852467 and SNP03_7.210477 which were absent in RiceVarMap2. For the targeted SNPs that were significant in a specific subpopulation, AUS, IND, TEJ or TRJ, the reference allele frequency ranged from 7.0% to 99.6% with five of the 21 reference allele frequencies for the targeted SNP being more than 90% among the accessions included in RiceVarMap2 or SNP-Seek. For the subpopulations where the reference allele frequency was above 90% or below 10% in these larger collections, the resistance allele is nearly fixed within the subpopulation, thus the SNP may not be useful for predicting resistance outside the context of RDP1.

### Tiller Number and Panicle Number QTL Identified from GWA Studies Across Subpopulations

Fifteen TN QTL and 14 PN QTL were identified (Fig. 2, Table 4, Additional file 1: Table S4) and the Manhattan and Q-Q plots are shown in Additional file 2: Fig. S3. All 14 PN QTL co-located with a TN QTL and were identified in the same panel(s) as the co-located TN QTL, with overlap between the PN and TN QTL evidenced by SNPs associated with TN and PN being interspersed with each other (Additional file 1: Table S4). Among the most-closely associated SNPs, selected as the targeted SNPs for each trait QTL based on their high -log_10_(*p*), which are low *p-*values (Table 4; Additional file 1: Table S4), one was identical (*qTN2-1* and *qPN2-1* among TEJ), and the other 13 were generally within 700 Kb (0.7 Mb) of each other (Table 4, Fig. 2, Additional file 1: Table S3). There were, however, three instances (*qTN4-2, qTN8-2* and *qTN11-2*) where the TN and PN targeted SNPs were more than 2.0 Mb apart, but all QTL had TN and PN peaks within 3.3 Mb. Based on this observation between the closely related TN and PN traits, for the present study, SNPs and QTL were considered to be in close proximity if they were < 3.3 Mb distant.

**Table 4:** QTL associated with tiller number (TN) or panicle number (PN) in the Rice Diversity Panel 1 (RDP1), arranged in chromosomal order to better indicate that 14 of the 15 QTL regions were associated with both TN and PN

QTL	Chr	Start of QTL region (bp)	End of QTL region (bp)	QTL size (bp)	Peak SNP (bp)^a^	− log_10_(*p*)	Trait^b^	Study location^b^	Panel^c^	Refer-ence allele^d^	Reference allele effect^d^	Candidate gene(s)		
												RAP ID^e^	Gene symbol(s)^f^ or name (s)	Citation
*qTN1-1*	1	18814513	23871743	5057230	18864513	5.4	TN	AR-gh	AUS	C	1.51	LOC_Os01g33240	*OsABCG33*	Bird et al. (2007), Yasuno et al. (2009), Zhao et al. (2019)
*qPN1-1*		17980650	20789712	2809062	18692039	7.1	PN	AR-gh	AUS	G	1.43	LOC_Os01g34970	*OsABCB2*	
↓												LOC_Os01g35030	*OsABCB3*	
*qTN1-2*	1	38561275	41357900	2796655	41275767	6.4	TN	AR-gh	AUS	C	1.83	LOC_Os01g69830	*OsSPL2*^g^	Dai et al. (2018)
*qPN1-2*		41677001	41952874	275873	41727001	5.0	PN	AR-gh	AUS	A	− 2.62	LOC_Os01g71310	*OsCKX4*^*g*^	Wang et al. (2019)
↓					41902874	5.4	PN	AR-gh	JAP	T	0.92	LOC_Os01g72330	*OsRR4*	Panda et al. (2018)
*qTN2-1*	2	663286	3898253	3234967	902313 3096122	5.1 6.9	TN	AR-gh	AUSTEJ	A C	− 3.06 –1.18	LOC_Os02g05840	*OsVIL2*^g^	Yoon et al. (2019)
*qPN2-1*		3046122	3208370	162248	3096122	5.9	PN	AR-gh	TEJ	C	− 1.18	LOC_Os02g04680	*OsSPL3*^g^	Liu et al. (2015)
*qTN2-2*	2	23111877	23211877	100000	23161877	5.4	TN	AR-gh	TEJ	C	− 2.15	LOC_Os02g38130	*OsNAC50*	A NAM, IRGSP-1.0, Kawahara et al. (2013)
*qPN2-2*		22525572	26480368	3954796	24663719	5.6	PN	AR-gh	TEJ	A	0.77	LOC_Os02g39920	*OsBIP135*	Fang et al. (2020)
↓												LOC_Os02g41450	*OsNAC51*	a NAM, IRGSP-1.0, Kawahara et al. (2013)
*qTN4-1*	4	12678105	15595957	2917852	13078293	7.3	TN	AR-gh	IND	G	− 1.66	LOC_Os04g23440	*OsMADS25*^g^	Zhang et al. (2018)
↓					14591830	5.8	TN	AR-gh	JAP	G	− 6.88	LOC_Os04g23910	*OsMADS82*^g^	Sui et al. (2016)
*qTN4-2*	4	21832919	28493892	6660973	21882919	7.2	TN	AR-gh	AUS	C	− 1.79	LOC_Os04g36070;	*OsRR1*^g^	Wang et al. (2019)
↓					28443892	5.6	TN	AR-gh	INDAUS	CG	− 1.85	LOC_Os04g39489	*OsAPP13*^g^	Wang et al. (2020)
*qPN4*	4	20986816	26514418	5527602	24460118	7.5	PN	AR-gh	INDAUS	A	− 1.12	LOC_Os04g46470	OsCCD7/*HTD1*^g^	Zou et al. (2006)
↓					26425281	8.4	PN	AR-gh	AUS	G	− 1.61	LOC_Os04g46580	*OsSPL7*^g^	Dai et al. (2018)
*qTN6*	6	27119407	27486520	367113	27058472	5.7	TN	AR-gh	AUS	T	− 2.64	LOC_Os06g44970	*OsPin2*^g^	Chen et al. (2012)
*qPN6*		27204808	27800000	595192	27254808	5.3	PN	AR-gh	AUS	A	− 1.02	LOC_Os06g45310	*OsSPL11*	Preston and Hileman (2013), Liu et al. (2015)
*qTN8-1*	8	8359495	9777576	1418081	9513920	5.7	TN	AR-gh	IND	A	− 1.91	LOC_Os08g15840	*OsXBO* *S35*	Tavakol et al. (2015), Zhang et al. (2019a, b)
*qPN8-1*		9586995	11777086	2190091	9636995	6.1	PN	AR-gh	IND	T	− 1.33			
*qTN8-2*	8	24971609	25071609	100000	25021609	5.5	TN	AR-gh	IND	C	2.10	LOC_Os08g39890	*OsSPL14/ipa1*^g^	Liu et al. (2019)
*qPN8-2*		25864572	27370635	1506063	27320051	5.7	PN	AR-gh	IND	C	0.94	LOC_Os08g41720	*OsPIN5b*^g^	Lu et al. (2015)
↓												LOC_Os08g42690	*OsXBOS252*	Tavakol et al. (2015), Zhang et al. (2019a, b)
*qTN10*	10	19312710	22648248	3335538	19373373	6.3	TN	AR-gh	AUS	C	2.81	LOC_Os10g35930	*OsPLIM2c*	Na et al. (2014)
*qPN10*		19536678	19636678	100000	19586678	5.9	PN	AR-gh	AUS	A	1.24	LOC_Os10g36703	*OsSAUR56*	Auxin-responsive, IRGSP-1.0, Kawahara et al. (2013)
↓												LOC_Os10g36710	CAMK gene	Ikeda et al. (2011)
*qTN11-1*	11	3999917	4144702	145880	4094702	5.5	TN	AR-gh	TRJ	A	0.79	LOC_Os11g06900	amidase family protein	IAA synthesis, Mano et al. (2010)
*qPN11-1*		3366987	4145797	777715	3416987	7.0	PN	AR-gh	TRJ	C	− 1.29	LOC_Os11g06820	*OsAUX5*	IRGSP-1.0, Kawahara et al. (2013)
↓												LOC_Os11g07700	*OsNAC126*	a NAM, IRGSP-1.0, Kawahara et al. (2013)
*qTN11-2*	11	12957650	17775288	4817638	14153306	5.9	TN	AR-gh	IND	C	1.65	LOC_Os11g25990	*OsIAGLU*^*g*^	Choi et al. (2012)
↓					17698549	5.4	TN	AR-gh	TRJ	A	0.89	LOC_Os11g29840	*OsLazy1*	an auxin transporter gene per IRGSP-1.0, Kawahara et al. (2013)
*qPN11-2*		13707162	18455763	4748601	17429259	5.1	PN	AR-gh	IND	G	− 1.62	LOC_Os11g30370	*OsSPL1* *9*	Liu et al. (2015), Dai et al. (2018)
↓					18405421	5.2	PN	AR-gh	AUS	T	− 2.10			
*qTN12-1*	12	2585066	5102002	1603575	2635066	6.5	TN	AR-gh	AUS	G	1.68	LOC_Os12g05590	*OsRCN3*^g^	Nakagawa et al. (2002)
*qPN12-1*		2654229	4029671	1375442	2714516	5.3	PN	AR-gh	AUS	A	1.16	LOC_Os12g05990	*OsNAC134*	NAM, IRGSP-1.0, Kawahara et al. (2013)
↓												LOC_Os12g07480	*OsTCP28*	branching factor, IRGSP-1.0, Kawahara et al. (2013)
*qTN12-2*	12	10383053	11837888	4823940	11787888	5.8	TN	AR-gh	IND	C	2.20	LOC_Os12g13720	*OsABCG49*	three other ABC transporters affect TN per Bird et al. (2007), Yasuno et al. (2009), Zhao et al. (2019)
*qPN12-2*		10394663	12121979	5158266	9285514	5.7	PN	AR-gh	AUS	C	1.48	LOC_Os12g17310	*OsBIP134*	Fang et al. (2020)
↓					12071590	5.7	PN	AR-gh	IND	C	1.33	LOC_Os12g21710	*OsZ-ISO*^*g*^	Liu et al. (2020)
*qTN12-3*	12	14477811	17557077	3079266	15793337	5.6	TN	AR-gh	AUS	G	− 1.63	LOC_Os12g27994	*OsDEC1*	per Itoh et al. (2012), alters auxin/CK
*qPN12-3*		14110118	17563794	3453676	16483239	6.2	PN	AR-gh	AUS	T	− 1.29	LOC_Os12g28270	*OsAH*	Rampey et al. (2004)
↓												LOC_Os12g29330	*OsNAC139*	a No Apical Meristem protein per IRGSP-1.0, Kawahara et al. (2013)

Tillers were counted in three replications of young plants 5- to 6-weeks old grown in the greenhouse, planted with repeated checks in an augmented design so that greenhouse location effect could be removed from RDP1 accession data. Panicle number was counted in the same replication 2 and 3 plants grown to maturity

^a^*O. sativa* SNPs are identified by their physical location based on the Os-Nipponbare-Reference-IRGSP-1.0 assembly (Kawahara et al. 2013)

^b^Tiller number (TN) was counted in three replications of 5- to 6-wk-old greenhouse grown plants; panicle number (PN) was evaluated using the same replications 2 and 3 plants grown to maturity. Best linear unbiased estimates (BLUEs) were calculated per replication, and across replications

^c^Panels are defined as the complete Rice Diversity Panel 1 (395) and subpopulation groups, *tropical japonica* (TRJ), *temperate japonica* (TEJ), *aus* (AUS) and *indica* (IND). The two *O. sativa* subspecies *Indica* (INDAUS) comprised of IND and AUS and *Japonica* (JAP) comprised of TEJ and TRJ. QTL were often identified in GWA-mapping of more than one population (e.g., in INDAUS and AUS); the table presents the results based on the least-complex population in which the QTL was found significant, except where inclusion of both INDAUS and AUS peaks validated the size of the overlapping *qTN4-2* and *qPN4*

^d^Reference allele based on the Os-Nipponbare-Reference-IRGSP-1.0 assembly (Kawahara et al., 2013). A negative allele effect at a TN or PN locus reflects a reduction in the tiller number or panicle number associated with the reference allele

^e^RAP ID is the Rice Annotation Project identification locus identified for the candidate gene

^f^Gene nomenclature followed the standardized nomenclature for rice genes used in *Oryzabase* (Yamazaki et al. 2010)

^g^Indicates a candidate gene that has been reported to have direct effect on tiller number per the cited study. All other candidate genes are less direct, e.g. are a member of a gene family in which a different member has been shown by the cited study to have a direct effect on TN, or a gene documented to have a direct effect on a hormone known to affect TN

Among the 15 TN QTL, nine were identified by GWA mapping as impacting TN and/or PN within AUS, five in IND, and only two each in TEJ and TRJ (Additional file 1: Table S4). This is consistent with our earlier observation that AUS had the widest variance for TN, followed by IND, with the TRJ having the narrowest variance as well as the lowest subpopulation average (Table 1). The reference allele frequencies at the targeted TN SNPs (Additional file 1: Table S5) were found to be similar in the same subpopulation(s) when compared between the RDP1 and RiceVarMap2 populations, indicating that the targeted SNPs identified among the RDP1 can also be useful for making QTL predictions beyond the RDP1.

While all 14 PN QTL overlapped with TN QTL, only five of the 18 ShB QTL overlapped with TN QTL (*qShB2, qShB4-2, qShB8-2, qShB11-3*, and q*ShB12*), with another three ShB QTL being considered as in close proximity with TN QTL because they included SNPs within 3.3 Mb of the edge of a TN QTL (*qShB4-4, qShB6-2*, and *qShB11-1*) (Fig. 2, Additional file 1: Table S3). Pivot table analysis of allele effects revealed favorable association between reduced ShB and increased TN was at seven of the eight overlapping or close QTL. Only at *qShB6-2* did pivot table analysis show that the allele associated with increased ShB resistance was unfavorably associated with a reduction in the TN and PN yield components. Thus, an increase in TN did not necessarily increase ShB severity. Further evidence discounting concerns of a detrimental association between TN and ShB, is the fact that none of the three QTL with the highest “-log10(p)” (or lowest *p-*value), in other words, the largest phenotypic effect on TN or PN (*qTN4-2, qTN10,* and *qTN12-2*) were closely linked with a ShB QTL.

## Discussion

### Increased Tiller Number Did Not Cause Increased Sheath Blight Severity

The primary purpose of mapping TN and PN along with ShB in the RDP1 was to evaluate if TN has a cause-effect relationship with ShB disease as seen in height, heading or culm habit with ShB. However, contrary to our initial hypothesis that increased TN would decrease airflow through the plant canopy, thus increasing ShB the same as erect culm habit does (Zuo et al. 2014) among the RDP1, we observed instead a negative rather than positive correlation between ShB and TN. Furthermore, for seven of the eight instances when ShB and TN QTL overlapped or were closely linked, the alleles associated with reduced ShB severity were determined by pivot table analysis to favorably increase TN, not decrease it. Although some TN and ShB QTLs were found co-located, TN was not found to be a causative confounding factor for ShB like height, heading date, and culm habit are, thus it was appropriate to not include TN as a covariate in the GWA mapping to identify ShB QTL not caused by an architectural attribute. It further indicates that it will be possible for breeders to breed for increased TN without necessarily decreasing ShB resistance.

### Colocalized Sheath Blight QTL Identified in GWA Studies and Biparental Populations

To validate the ShB GWA-QTL identified in this study we surveyed both GWA and biparental QTL mapping studies and summarized the colocalized QTL in Additional file 1: Table S6. Of the seven ShB GWA studies surveyed, four evaluated ShB reaction using the microchamber method (Chen et al. 2019; Jia et al. 2012; Rosas et al. 2018; Sun et al. 2014), Oreiro et al. (2020) evaluated the detached main tiller at the maximum tillering stage of plants grown in a screenhouse, Wang et al. (2021) scored tillers grown under field conditions at the seedling, maximum tillering and booting stages, and Zhang et al. (2019b) measured lesion length on tillers of field grown plants. Overall, only the *qShB9* ShB QTL identified in this study was not validated in at least one of these GWA studies.

Of these studies, Chen et al. (2019) was the most closely aligned with ours because 299 RDP1 accessions were evaluated, and the method was similar to the microchamber method. The seedlings were inoculated with the *R. solani* strain YN-7 from Yangzhou University, China and rated according to Jia et al. (2007). GWA mapping with 44,000 SNPs identified *qSB-3* and *qSB-6* which were co-located with *qShB3-3* and *qShB6-2*, respectively, described in this study. Also, *qSB-7* was about 1.0 Mb from *qShB7* reported in this study.

Jia et al. (2012) previously mapped 10 ShB GWA-QTL in the RMC (217 accessions) based on microchamber ShB ratings collected in AR using the same *R. solani* isolate (RR0140-1). Each QTL was tagged with a single SSR marker. The present GWA mapping with 3,200,320 SNP genotypes for 167 RMC *O. sativa* accessions (Additional file 1: Table S7) identified three or more significantly associated SNPs within 6.0 Mb for six of the 10 ShB-QTL tagging SSR markers reported by Jia et al. (2012). In two cases, the presently identified *qShB2-mc* and *qShB4-mc* were less than 1.0 Mb from the presently identified RDP1 GWA-QTL *qShB2* and *qShB4-3*, respectively (Additional file 1: Table S7). Five RMC GWA-QTL which were newly detected in the present analysis and four of these (*qShB7-mc*, *qShB10-mc*, *qShB11_2-mc* and *qShB12-mc*) were co-localized with RDP1 ShB GWA-QTL, *qShB7*, *qShB10*, *qShB11-2* and *qShB12*, respectively, providing multi-population validation of these ShB-QTLs.

The ShB QTL identified in biparental populations that are near (approximately 3 Mb) or co-localized with the 18 RDP1 ShB GWA-QTL we report, as ascertained from the Mb position of the peak or flanking markers are summarized in Additional file 1: Table S6. Only one of the 18 RDP1 ShB QTL, *qShB4-1*, was not near a previously reported biparental QTL. Across the 17 GWA-QTL colocalized with biparental QTL, *qShB9* was validated by colocalized QTL reported in ten different biparental populations; the *qShB3-3*, *qShB6-1*and *qShB7* were confirmed by colocalization in four to six different populations; and *qShB4-3* and *qShB11-1* were substantiated in three different populations. The remaining eleven GWA-QTL were each supported by ShB QTL identified in one or two different biparental populations (all detailed and cited in Additional file 1: Table S6).

### Candidate Genes in the Sheath Blight GWA-QTL Regions

To date, only three ShB QTL have been fine-mapped, *qSB-9*^*TQ*^ (Zuo et al. 2014), *qSB-11*^*LT*^ (Zuo et al. 2013) and *qSBR11-1* (Channamallikarjuna et al. 2010). Zuo et al. (2014) reported 12 candidate genes potentially associated with quantitative resistance to necrotrophic pathogens like *R. solani* in the chr. 9 QTL, *qSB-9*^*TQ*^ region (21.37–21.52 Mb) which is included in the *qShB9* GWA-QTL we report (Table 3, Additional file 1: Table S4). Also, the *qSB-11*^*LT*^ on chr. 11 (4.78 to 4.87 Mb) was about 4.1 Mb from *qShB11-1*. Additionally, of note, the *R. solani* phytotoxin sensitivity gene, *Rsn1*, on chr. 7 at 18.1 Mb (Costanzo et al. 2011) was more than 4 Mb from *qShB7*.

To further explore candidate genes in the 18 ShB GWA-QTL (Table 3), we searched the merged database file (described in the “Candidate Gene Identification” Methods section), for components of the jasmonic acid pathway because generally the jasmonic acid-mediated signaling induces resistance against the necrotrophic fungi like *R. solani* (Browse 2009; Glazebrook 2005; Kunkel and Brooks 2002), as well as, to *Magnaporthe oryzae* which causes rice blast disease, *Xanthomonas oryzae* pv. *oryzae* (*Xoo*) which causes bacterial blight, and to abiotic stresses like heat, cold and salt. Closely related to this pathway are the jasmonates which include the biologically active intermediates of this pathway plus jasmonic acid derivatives. We also considered several other molecular interactions between the rice plant and *R. solani*, including enzymes targeting the plant or fungal cell wall components, transport across the cell wall, interactions with WRKY transcription factors, regulators of programed cell death, as well as receptor-like cytoplasmic kinases (RLCKs) and ribosomal proteins which affect crucial plant developmental processes summarized by Li et al. (2021) and Molla et al. (2020). Across the 18 ShB QTL regions (Table 3), we identified 26 candidate genes associated with the jasmonic acid pathway or response to *R. solani* and 13 candidate genes responsive to *M. oryzae*, *Xoo* or the abiotic stresses cold, heat or salt.

Related to the jasmonic acid pathway, we identified *OsAlba7* (acetylation lowers binding affinity protein 7) in the *qShB9* region, allene oxide cyclase (*OsAOC*) (Shimizu et al. 2013; Yoeun et al. 2018; Yu et al. 2020) and *TIFY10A* in the *qShB3-2* region, and the duplicate *TIFY10B* in *qShB7* QTL (Ye et al. 2009). *TIFY10A* and *TIFY10B* are part of the *JASMONATE-ZIM DOMAIN* (*JAZ*) gene subfamily which produces proteins that are key regulators of the jasmonate hormonal response, and these genes are responsive to wounding, abiotic stress and jasmonic acid (Shimizu et al. 2013; Ye et al. 2009).

In the *qShB3-2*, the targeted SNP at 15.55 Mb was in the region of four of the eight rice metacaspase (*OsMC*) genes (Wang and Zhang 2014). Metacaspases are cysteine-dependent proteases and important regulators of programmed cell death during development and stress responses in plants. Members of the *OsMC* family displayed differential expression patterns in response to abiotic and biotic stresses, including *R. solani* and stress-related hormones like jasmonic acid (Huang et al. 2015).

Chitin is the major component of fungal cell walls. An immune response is initiated in plants when chitin is recognized by the pattern recognition receptor *CEBiP* and its coreceptor, *OsCERK1* on the cell membrane (Li et al. 2021; Shimizu et al 2010). *OsCERK1* maps to the *qShB8-2* region (Carotenuto et al. 2017). Additionally, *OsRacGEF2* which maps to the *qShB9* region, forms a complex with *OsCERK1* to enable transport from the endoplasmic reticulum to the cell membrane and the formation of a stable immune complex (Akamatsu et al. 2015). Similarly, *CHITINASE 6,* a candidate gene for *qShB2* (Table 3)*,* is proposed to encode a pathogenesis-related protein(s) having antifungal activity, making it important for disease resistance (Nakazaki et al. 2006).

Transport across the cell wall is another important aspect of the rice plant’s defense. Acyl-CoA binding proteins (ACBPs) have been reported to play a role in plant stress, including defense against pathogens. Six ACBPs were identified in *O. sativa*, including *OsACBP5* which appears to participate in the extracellular transport of acyl-CoA esters and/or other lipids and is up regulated in response to *R. solani* infection and wounding (Liao et al. 2020; Panthapulakkal Narayanan et al. 2019). Also, the ATP binding cassette (ABC) transporters are responsible for the ATP-powered translocation of many substrates across membranes. Of note, two of the three ABC transporters which were induced in response to *R. solani* (Oh et al. 2021) namely *OsABC9* and  OsABC12, were located in the GWA-QTL *qShB9* and near *qShB12*, respectively.

Lastly, several WRKY transcription factors are important components of plant defense against pathogen infection. Of the seven WRKY transcription factors expressed in response to *R. solani* infection summarized by Li et al. (2021), only *OsWRKY33* was in a ShB GWA-QTL region. *OsWRKY33* was more highly expressed in the resistant cultivar and is required for resistance to necrotrophic pathogens (Shi et al. 2020). Additionally, Pooja et al. (2015) reported expression of *OsWRKY102* was high upon inoculation with *R. solani*, compared to the uninoculated control.

### Candidate Genes in the Tiller Number GWA-QTL Regions

In stark contrast to sheath blight with few fine-mapped genes, there are numerous genes for which altered sequence or expression have been directly associated with altered tiller bud initiation, tiller elongation, and/or panicle number to be considered when identifying candidate genes underlying TN and PN QTL. Many of the known TN genes were discovered and documented through the study of natural or induced mutations identified as having notably fewer tillers or more numerous tillers than their wildtype counterparts (e.g., the *reduced culm number* (*RCN*), *monoculm* (*MOC*), and *high tillering dwarf* (*HTD*) genes). Seven of the 15 TN GWA-QTL regions include one or more genes for which mutation or altered expression were directly linked with altered tiller number or development and are annotated in Table 4, which also cites the studies documenting direct connection with TN. The additional candidate genes listed in Table 4 are either in a gene family for which a different member has been reported to directly impact tiller development (e.g., the ABC transporters for *qTN1-1*), or is a gene documented to impact synthesis or accumulation of a hormone that is known to impact tiller elongation or bud production, such as auxin, cytokinin, brassinosteroids, strigolactones (reviewed by Hussien et al. 2014; Liang et al. 2014). For example, *qTN1-1* does not contain a gene previously documented to affect TN, but it is possible that one or more of the three ABC transporters in this QTL region (Table 4) are affecting TN in that three other rice ABC transporters are known to impact rice TN (Bird et al. 2007; Yasuno et al. 2009; Zhao et al. 2019).

The five TN QTL that overlapped with ShB QTL all showed favorable association between increased TN and decreased ShB severity, based on pivot table evaluations. It is possible that these instances of QTL overlap are due to favorable linkage of different genes, one affecting TN and another affecting ShB response. However, such QTL overlaps could also be due to pleiotropic effects of a single gene because auxin (Denance et al. 2013) and cytokinin (Chanclud et al. 2016, Costanzo et al. 2011) can impact not just tiller development, but also fungal pathogen virulence. Further study would be required to rule out that the QTL overlaps are not due to one or more genes having pleiotropic effect on both TN and ShB by impacting auxin or cytokinin concentration or availability. One such example among the TN candidate genes in the region where *qTN12-3* and *qShB12* overlap is *Auxin Homeostasis* (*OsAH,* Rampey et al. 2004) (Table 4). A second example would be the *DECUSSATE 1* (*OsDEC1)* gene in this same overlapping ShB-TN QTL region, with *DECUSSATE* genes known to alter auxin/cytokinin ratios (Itoh et al. 2012). Each of the remaining four TN QTL regions that overlap with ShB QTL (*qTN2-2, qTN4-1, qTN8-2*, and *qTN11-2*) also contain genes known to affect auxin synthesis or transport (Table 4).

### High Panicle Number Predicted from Early Tiller Number

The high frequency of colocalized PN QTL with TN QTL corroborates TN as a yield component via PN. Furthermore, the colocalization of QTL shows that genotypes with increased PN can be indirectly detected and selected based on increased TN observed in young plants (5- to 6-wk age, mid-tillering stage), as used in this study. Based on biparental mapping and selection studies, Pinson et al. (2015, 2016), and Pinson and Jia (2016) first proposed this as a cross-progeny breeding strategy wherein indirect selection for PN can occur prior to flowering so that subsequent crossing can be accomplished in the same generation, thus accelerating breeding progress. The present RDP1 results extend the utility of indirect selection for PN based on young plant TN by showing it can also be effective among genetically diverse accessions.

Detection of TN gene effects in young plants would also benefit the further analyses that would be required to determine which, if any, of the candidate genes are responsible for the TN and PN QTL. For example, *qTN8-2* contains two well documented TN genes, the *ideal plant architecture 1* (*ipa1*) (Liu et al. 2019) and *OsPIN5b* (Lu et al. 2015). Similarly, *qTN4-1* is a long QTL region, extending 7.5 Mb, and encompassing four genes known to affect TN: *OsRR1* (Wang et al. 2019), *OsAPP13* (Wang et al. 2020), *OsCCD7/HTD1* (Zou et al. 2006), and *OsSPL7* (Dai et al. 2018). It may be that more than one of these known genes underlie the lengthy *qTN4-1* QTL. Researchers may have more interest, however, in studying the eight TN QTL that do not contain genes previously known to impact TN because these QTL offer greater opportunity to discover novel TN genes. For example, Jiang et al. (2019) also identified a TN QTL in the present *qTN1-1* region using a different rice diversity panel, thus validating *qTN1-1* in multiple populations though it does not contain a previously known TN gene. Similarly, the *qTN2-2* QTL region which spans from 22.5 to 26.4 Mb on chr. 2 does not contain a known TN gene though six prior rice studies mapped TN QTL to this region.

### Selection of Sheath Blight Resistant Accessions as Potential Parents for Breeding

The RDP1 phenotypic data plus genotypes at the identified ShB GWA-QTL are useful for identifying subsets of resistant germplasm based on various selection schemes. For example, 49 accessions had NC-field ratings ≤ 4.5 combined with AR-field ratings not > 3.5 (Additional file 1: Table S8). This selection criterion uses a more relaxed threshold for NC-field due to its higher disease severity and includes lines that were not evaluated in AR-fields due to red pericarp if they performed well in NC-fields. Even though expected from the strong ShB-height correlations (Table 2), selections based on field data are unfortunately considerably taller, being on average 22 cm taller than the RDP1 average in NC, and 27 cm taller than the AR average. Maturity was also delayed 3 to 6 days in plants selected on their field resistances alone. The microchamber DI is reportedly less biased toward tall plants, and indeed the 20 accessions having AR-DI ≤ 3.5 and NC-DI ≤ 6.7 showed lower average height increase (9 cm in NC, 5 cm in AR), and maturity was delayed only 2 to 3 days. Making phenotypic selections based on low ShB ratings in both field and DI selections culls the accessions down to eight, four from IND plus one each from AUS, TEJ, TRJ and admixed-IND accessions.

Considering the geographic origins of accessions containing desired traits can reveal “hotspots”, or regions of the world where desired genes are more concentrated. Interestingly, of the four accessions tagged with SNP data in RDP1 that originated from the island of Madagascar, three were selected as resistant based on field or DI ratings, with two of them being selected based on both field and DI resistance. The fourth Madagascar accession did exhibit low ShB severity in NC fields, but slightly exceeded the DI selection criteria. This suggests that Madagascar may be a hotspot for ShB resistance alleles. However, the accessions from Madagascar are taller than desired, ranging in height from about 127 to a very tall 182 cm (Additional file 1: Table S8), while even non-semidwarf modern cultivars in the USA are less than 120 cm (Additional file 1: Table S8), raising the question of how many of the height-independent ShB QTL these relatively resistant RDP1 accessions contain.

Unfortunately, based on pivot table analysis, many of the ShB QTL were not effective in all subpopulations (see Additional file 1: Table S8) making it difficult to determine the number of R-alleles present in each RDP1 accession, with genotypic predictions of R alleles being particularly unreliable for the admixture accessions that fell between the subpopulations. Even so, predictions were made per QTL per subpopulation as described in more detail in Additional file 1: Table S8. In brief, accessions having R-associated alleles for a particular QTL were coded as R = 1, as opposed to S = 0. If the pivot table analysis indicated a particular target SNP was not applicable to a particular subpopulation or set of admixed accessions, they were coded as ‘na’. After coding each RDP1 accession based on one or more target SNPs in the 18 ShB GWA-QTL, the sum of R alleles per RDP1 was calculated (Additional file 1: Table S8). The RDP1 accessions were thus estimated to contain from 0 to 11 R-alleles, with a population average of 6.5 ± 2.0 alleles. One of the accessions from Madagascar consistently selected as ShB resistant was a TRJ, which also was selected for containing a high number of R alleles (9) among the TRJ accessions with an overall average of 6.8 R alleles (Additional file 1: Table S8). The other three accessions from Madagascar were admixtures, making their R allele estimates less reliable but worthy of further investigation. The Madagascar accessions were estimated to contain 6, 8, 8, and 9 R alleles each, with all four accessions being R at *qShB2, qShB6-2,* and *qShB7*, and the three Madagascar accessions that were selected for their relative resistance under AR and NC field or DI conditions also contain the R allele at *qShB1*, *qShB3-2, and qSh3-3,* increasing confidence and breeder interest in these particular ShB QTL.

To further assess the ShB GWA-QTL, we evaluated shifts in trait averages after making selections based on the marker-predicted number of R-alleles per accession. Selections for the most R-alleles were made per panel because several QTL were not applicable outside of IND, AUS, and INDAUS (Additional file 1: Table S8). Selections were made only in the AUS, IND, TEJ and TRJ panels because the ARO panel was small and the R-allele predictions were especially confounded in the admixed accessions by the different effectiveness of the various QTL in different panels. Selection of accessions having numerous R-alleles per their subpopulation identified approximately 25% of the AUS, IND, TEJ and TRJ accessions (shown in Additional file 1: Table S8). When trait means were compared across the selected versus unselected accessions per panel (Additional file 1: Table S9), selection for increased R alleles was found to decrease ShB severity for most subpopulations and most studies (AR and NC, field and DI), with the AR-field ShB ratings decreasing by as much as 1.2 units among the TRJ. Selections based on the number of R-alleles were, overall, less effective for NC-field than for AR-field. Selection for numerous R-alleles was also less effective among the TRJ than the other subpopulations, with a notable reduction in ShB detected only in the AR-field. Even so, across all the studies and panels, including the less affected TRJ panel, selection of accessions containing more numerous R-alleles decreased ShB rating by an average of 0.6 rating units, indicating that the selections made solely on genotypes at the QTL targeted SNPs were effective in general.

This study used both microchamber-DI data, and field ShB data with plant height and heading as covariates in the GWA analyses with the intention of identifying ShB QTL that were not confounded by tall height and late heading, thus more likely to impart disease resistance through physiological or biochemical modification of host–pathogen interactions, e.g., the many candidate genes listed in Table 3. Even so, selections based on marker-predicted R-alleles among the IND increased height by 13 cm, and delayed heading by 11 days based on AR-field trait data (Additional file 1: Table S9). In contrast, among the AUS subpopulation, which is the tallest of the subpopulations, average height showed an insignificant decrease of 1 cm among the marker-selected AUS, where the average delay in heading was also insignificant. Even though some R-alleles were colocated with TN QTL, selection for more numerous R-alleles did not significantly alter TN in any subpopulation.

## Conclusion

Of the 18 ShB GWA QTL reported in this study, *qShB4-1* was a newly identified QTL and the other 17 QTL were located within or near previously reported QTL identified in biparental mapping studies. It should be noted that the QTL regions reported in this study were more precisely defined than most of the previously reported QTL, and the RDP1 accessions may be a source of novel alleles even for previously reported QTL. Including plant height and heading as covariates in the GWA analyses successfully removed these confounding effects and revealed ShB QTL not caused by tall height and late heading. Co-location of a heading QTL with *qShB6-1* was found, but the difference in maturity was not consistent between subpopulations, thus it suggests the utility of including traits as covariates in GWA analysis to identify unconfounded QTL.

Even though PN and TN QTL were co-located with five of the ShB QTL, the alleles that decreased ShB severity were found associated with increased TN and PN, not with decreased TN as was hypothesized if increased TN was causing increased disease development. This indicates that PN and TN do not directly affect pathogen growth or spread and can be improved by breeders along with selection for increased ShB resistance.

Co-location with previously reported QTL and/or candidate genes increases confidence in the ShB, TN, and PN QTL identified in this study. The ShB QTL were further verified when selection based on phenotypes and the converse selections made per subpopulation based on QTL genotypes both documented that ShB resistance improved with an increase in R alleles. From a combination of phenotypic and genotypic selections, 19 accessions were identified as containing numerous R alleles along with exhibiting resistance in both AR and NC field trials, and six QTLs common among accessions from Madagascar, a putative hotspot for ShB resistance, were noted. Rice breeders can utilize these accessions and SNPs to develop cultivars with enhanced ShB resistance along with increased TN for improved yield potential.

## Material and Methods

### Description of the Rice Collections

The Rice Diversity Panel 1 (RDP1) is a collection of 424 purified, homozygous rice accessions representing the broad range of genetic variation within *O. sativa* (Eizenga et al. 2014). The accessions include both landrace and elite rice cultivars classified into the five subpopulation groups, IND (96 accessions), AUS (61), TRJ (108), TEJ (110) and ARO (16). The remaining 33 accessions were classified as admixtures because they shared < 60% ancestry with a single subpopulation.

The USDA-ARS Rice Minicore (RMC) collection includes 202 purified, homozygous *O. sativa* accessions identified as IND (68 accessions), AUS (38), TRJ (33), TEJ (34) and ARO (6). The remaining 23 accessions were an admixture of two or more subpopulation groups (Agrama et al. 2009; Schläppi et al. 2017). Both the RDP1 and RMC accessions were obtained from the USDA-ARS Genetic Stocks-*Oryza* (GSOR) collection located at the USDA-ARS Dale Bumpers National Rice Research Center (DBNRRC), Stuttgart, Arkansas USA (www.ars.usda.gov/GSOR).

### Evaluation of Sheath Blight Disease and Associated Plant Architecture Traits

RDP1 accessions were evaluated for reaction to ShB disease at the DBNRRC in the greenhouse using the microchamber method as described by Jia et al. (2007) and in the field over two growing seasons [May to September 2016 (AR16) and 2017 (AR17)]. As part of the microchamber method, four-week-old seedlings were inoculated with a potato dextrose agar block containing actively growing *R. solani* mycelia, thus the ratings were not confounded with plant height or heading. For this study, the experiment was arranged in a randomized design and replicated three different times. The plants were inoculated with *R. solani* isolate RR0140-1 which produced consistent symptoms on reference cultivars (Jia et al. 2007) and was confirmed as a true isolate of *R. solani* with molecular data (Wamishe et al. 2007). The seedlings were rated for ShB response by measuring the culm length and the length of the disease lesion from the base of the plant. The disease index (DI) was calculated by dividing the culm length into the lesion length and multiplying by 9 as described by Jia et al. (2007). The check cultivars were Lemont as highly susceptible, Cocodrie as moderately susceptible, and TeQing and Jasmine85 as moderately resistant and also are in the RDP1. Six or seven sets of the four checks were included with each replication and 401 RDP1 accessions were evaluated for ShB reaction.

In Nanning, China (NC) 412 RDP1 accessions were evaluated for ShB disease in the greenhouse by means of the microchamber method using the local isolate, *R. solani* AG1-1A, which was identified in Guangxi Province (Hu et al. 2010) and found to be the most virulent of the fungi associated with rice sheath blight disease. The evaluation was replicated three times and three sets of the two check cultivars, Lemont and TeQing were included in each replication. The DI was calculated for each accession or check cultivar evaluated as described above (Jia et al. 2007).

### Sheath Blight Field Evaluations

To prevent outcrossing into commercial fields, accessions with a red pericarp could not be planted in the field in Arkansas, therefore only 347 RDP1 accessions were evaluated for ShB during the 2016 growing season (AR16), and 353 (five additional non-red pericarp accessions) during 2017 (AR17). The accessions were planted in 3-row plots arranged in a randomized complete block design with two replications per year. The drill-seeded three-row plots were 1.52 m long with 30.5 cm between the rows and approximately 3 g seed per row to ensure a dense plant stand. Seven sets of the aforementioned four check cultivars were included with each replication. A dense six row border was planted around each replication in 2016. In 2017, the same field design was used but there was no border because the seed for the border had poor germination.

A permanent flood was applied to fields approximately 30 days after planting. Plants were inoculated at panicle initiation, the R0 stage (Counce et al. 2000), with a mixture of *R. solani* mycelia growing on a medium of crushed maize (*Zea mays* L.) and ryegrass (*Lolium perenne* L.) prepared as described by Liu et al. (2013). Inoculum of the isolate RR0140-1 was spread at the base of each plant, with 30 g of inoculum spread over each progeny row. After this, the inoculum floats on the flood water and infects the plants when it comes into contact with the rice stem and subsequently the disease symptoms progress up the plant canopy (Lee and Rush 1983; Webster and Gunnell 1992). ShB severity is rated based on the proportion of above-water vegetation showing ShB symptoms thus, the field rating system can be confounded by plant architecture especially height, heading, culm habit and tillering. The progeny plots were rated for ShB reaction at the late R6 to early R7 stage (Counce et al. 2000) on a 0 (no disease), 1 (10% infected, etc. to 9 (≥ 90% infected, nearly dead) scale (Marchetti and Bollich 1991) with each unit of the scale representing the percentage of vegetative plant tissues above the water level that exhibited disease lesions. When the disease reached the flag leaf blade, it was rated “9” denoting it was at least 90% more infected.

Three agronomic traits, days to heading, plant height and culm habit were collected from the field experiment. Days to heading was calculated as the number of days required for 50% of the plants within a plot to have at least one tiller at anthesis. At maturity, the plant height (cm) of three plants in each row was measured from the base of the plant to the tip of the tallest panicle excluding the awn. In AR17, culm habit was estimated as visual observation from three plants in a row at the milky dough stage on a 1 to 5 scale having 1 (erect), 2 (intermediate), 3 (open), 4 (spreading) and 5 (procumbent) (IRRI 2002).

In Nanning, China, 418 RDP1 accessions and two check cultivars, Lemont and TeQing, were included in the transplanted field test during 2018 (NC18). For transplanting, 50 seeds of each entry were placed in a paper bag, soaked for 24 h in water, and subsequently placed in a tray on a wet paper towel and covered with a wet paper towel to maintain the humidity. Once the seed had germinated, it was planted in a 40 cm × 40 cm cell filled with soil and flooded with water. When the seedlings reached the four-leaf stage, 30 healthy seedlings were selected for transplanting to the field. The plot for each entry within each replication had three rows of ten plants each. The 10 plants were 13.2 cm apart within the row and there was 23.1 cm between the rows in the plot. Three sets of the two check cultivars, Lemont and TeQing were included in each replication. The plots were arranged in a randomized complete block design with three replications following standard field planting and management practices. The inoculation and rating followed the same method except the aforementioned local *R. solani* isolate AG1-1A was used (Hu et al. 2010). Days to heading and plant height were taken as described in Arkansas.

### Statistical Analyses of Sheath Blight Data

The generalized linear mixed model (GLIMMIX) procedure in SAS version 9.4 (SAS Institute Inc., 2012) was used to perform ANOVA on the data. Least square means (LSmeans) were based on the GLIMMIX procedure, with repeated checks or RDP1 accession as fixed effects and replication as a random effect. For the Arkansas field trials combined over years “ARall” the model considered year, replications nested within year, year × check, and RDP1 accession as random effects. Checks were included in each replicate block to control for field variability. To obtain the best linear unbiased predictions (BLUPs) of the RDP1 accessions, the model was revised to include the four check accessions and RDP1 accessions as fixed effects, and RDP1 accession as a random effect. A normal distribution was used by GLIMMIX for all traits evaluated in the field and microchamber. Based on these analyses, LSmeans were used for determining the summary statistics calculated in JMP 14 (SAS Institute Inc. 2018) and BLUPs were used for the GWA analyses. After reviewing the correlations, regression analyses and means comparisons within country as well as combined across Stuttgart, Arkansas, USA (AR) and Nanning, China (NC), it was decided to not combine data across countries, so that subsequent GWA mapping could clarify both differences and similarities between the countries.

### Use of Sheath Blight Ratings to Classify Accessions as Resistant or Susceptible per Study

Cultivars receiving an average ShB response rating ≤ 3 are often considered as resistant (R), while those with ratings > 6 are considered susceptible (S) (Chen et al. 2019; Li et al. 1995; Marchetti and Bollich 1991).  For this study, ShB ratings calculated across replications by country for the field (ARall, NC18) and microchamber (AR-DI, NC-DI) studies, were used to classify the RDP1 accessions into four categories: accessions with ShB rating ≤ 3 were considered resistant (R), those with ShB between 3.0 and 4.5 were classified as moderately resistance (MR), ShB from 4.5 to 6.0 were moderately susceptible (MS), and those with ShB > 6 were classified as susceptible (S).

### Evaluation of Tiller Number and Panicle Number per Plant

Tiller number (TN) and panicle number (PN) were evaluated per plant using planting and growth methods modeled after those used in multiple prior rice TN QTL studies (Barnaby et al. 2019; Pinson et al. 2015; Pinson and Jia 2016), conducted in greenhouses at the DBNRRC. Greenhouse growth allows control over several key factors known to alter tillering rates, such as planting depth, plant spacing, water depth, air temperatures, and day length. Three independent replications, each comprising one pot per RDP1 accession plus 25 pots each of two check cultivars, Zhe733 (PI634573) and Presidio (PI636465), were planted on April 7, July 2, and September 24, 2015. Each replication was planted in a single greenhouse where halogen lights maintained a minimum daylength of 14 h, and air temperatures were maintained between 26 and 39°C.

Six seeds were planted per pot, then thinned to two plants per pot at 11 to 13 days after seeding (approximately the 2-leaf stage). Pots (15 cm diameter, 18 cm deep) were filled to 14 cm with a soil mixture consisting of 3 parts (v/v) locally obtained Dewitt silt loam topsoil (fine, smectitic, thermic Typic Albaqualf; National Cooperative Soil Survey, 2014) that was steam sterilized before being thoroughly mixed in a cement mixer with one part commercial soil conditioner (Scotts Premium Humus and Manure, Scotts Company). Soil in pots was wetted thoroughly with fertilizer solution (4 g L^−1^ Jack’s Professional 20–20–20 N–P–K [J.R. Peter], plus 0.17 g L^−1^ iron chelate [Sequestrene 330 Fe, 10% Fe, Becker Underwood]). Six seeds were placed per pot, covered with 1.5 cm soil mixture, and wetted from above with fertilizer solution. Placement of pots into larger tubs (1.2 m × 2.4 m × 0.3 m deep), and holes in the bottoms of the pots, allowed soil to remain saturated by standing pots in 3–7 cm water, and approximately 50 mL of fertilizer solution was added to the top of each pot weekly.

The 422 pots per replication containing RDP1 were divided among 5 large tubs. Each replication was planted in an augmented design, with each tub containing five pots of each check cultivar: Zhe733 a high-tillering cultivar from China (Pinson and Jia 2016), and Presidio a US cultivar of moderate tillering ability. With different planting dates, plants were expected to grow at different rates between replications. Therefore, the plant growth stage of the high-tillering Zhe733 check was used, rather than calendar days, to standardize the TN observations between replications per plant growth stage. The total number of stems (TN) on each check plant was monitored twice weekly starting at two weeks after seeding. The TN of all RDP1 plants was collected at two points, once when the 50 Zhe733 plants in that replication reached an average TN ≥ 3 (early tillering), and again at later tillering when the Zhe733 TN average reached ≥ 5. Based on these physiological growth stages, the early TN counts occurred at 3, 3 and 4 weeks after seeding of replications 1, 2 and 3, respectively, and the later TN counts were collected at 5, 5 and 6 weeks. Across replications, the Presidio check plants had TN averages of 1.3 (with most plants having only the main culm stem) and 3.2 at early and later tillering, respectively. Replications 2 and 3 were grown to maturity, at which time the number of panicles per plant (PN) were counted.

The repeated checks were used to identify and remove greenhouse location effects on TN and PN from the RDP1 data as part of the augmented design. Analysis of variance determined that tub effects and the pot’s distance from the wall of greenhouse cooling pads were significant. RDP1 data were therefore adjusted by including these factors, and their tub × distance interaction, as fixed effects in the calculation of best linear unbiased estimates (BLUEs) per RDP1 per replication. BLUEs were calculated using GLIMMX (SAS Institute Inc. 2012) to calculate studentized residuals from overall replication means, and spatially adjusted RDP1 values were calculated by adding the residuals to the replication means. To calculate RDP1 BLUEs across replications, replication and tub nested within replication were considered as random effects, and distance from the cooling wall per replication and RDP1 accession were fixed effects. GWA analyses were run using TN data collected at the very early (3- to 4-week stage) and at the mid-tillering stage (5- to 6-week stage). The later TN dataset identified all the QTL identified using the very early TN data, and more. Therefore, results just from the second TN count per replication are discussed further and presented in Tables.

### Statistical Evaluation of the Trait Means and Correlations

For all ShB traits, days to heading, plant height and culm habit, LSmeans were used for subsequent statistical analyses and for TN and PN, BLUEs were used for both the statistical analyses and GWA mapping. Pearson’s correlations and regression analyses were conducted using SAS JMP 14 (SAS Institute Inc. 2018) for pairwise analysis of relationships between traits. Tukey–Kramer multiple mean comparison tests were used to detect differences among subpopulation or location means.

### Genome-Wide Association (GWA) Mapping Panels 

#### Rice Diversity Panel 1 (RDP1)

Recently, Wang et al. (2018) expanded the genotyping of the RDP1 accessions to 4,829,392 SNPs through imputation with data from the 3 K RGP (Li et al. 2014) to create the Rice Reference Panel (RICE-RP). The RICE-RP was downloaded from the European Variant Archive (https://www.ebi.ac.uk/ena/data/view/PRJEB26328) and the imputed genotypic data of 395 RDP1 accessions were selected for this study. To capture the genetic diversity specific to subpopulations, seven panels were created. All panels were filtered from the full 395 unfiltered genotype set independently to exclude heterozygous markers and markers with a minor allele frequency less than 5%. The first panel “395” or “RDP1” was composed of 395 accessions with 3,463,224 SNPs and contained all five rice subpopulations; IND, AUS, ARO, TRJ, TEJ and the admixtures. For the panels representing individual subpopulations, IND (83 accessions) had 2,278,953 SNPs, AUS (55 accessions) had 2,114,284 SNPs, TRJ (100 accessions) had 1,431,019 SNPs and TEJ (97 accessions) had 881,628 SNPs. In the *Indica* subspecies, the INDAUS panel included both IND and AUS (138 accessions) with 3,016,200 SNPs and for the *Japonica* subspecies, the JAP panel included TRJ and TEJ (197 accessions) with 1,445,489 SNPs. ARO had a limited number of accessions (14), thus was only included in the 395 panel.

For GWA mapping, BLUPs were used for the ShB traits, days to heading, plant height and culm habit and BLUEs for TN and PN. As plant height and days to heading are known to be confounding factors for field sheath blight ratings (Nelson et al. 2012; Srinivasachary et al. 2011), they were used as covariates during GWA mapping of both AR and NC field ShB data.

#### Rice Minicore (RMC) Collection

As previously mentioned, the RMC was evaluated for ShB reaction and GWA mapping conducted with 154 SSR markers and one InDel (Jia et al. 2012). Subsequent resequencing made high quality SNP genotypes publicly available (Chen et al. 2014; Wang et al. 2016). To take advantage of the much denser genotyping, GWA mapping was conducted using the previously published ShB LSmeans (Jia et al. 2012) and the SNPs obtained from resequencing (Wang et al. 2016), filtered as described by Huggins et al. (2019).

As with the RDP1, multiple panels based on subpopulation and subspecies were created to capture QTL that are specific to each group. The panel “All” contained 167 MC accessions including all five rice subpopulations as well as admixtures and had 3,200,320 SNPs. For the subpopulation panels IND (58 accessions) had 1,748,381 SNPs, AUS (30 accessions) had 1,630,989 SNPs, TRJ (28 accessions) had 1,044,932 SNPs and TEJ (30 accessions) had 680,948 SNPs. For the subspecies, INDAUS included 89 accessions [58 IND, 30 AUS and one *indica* (INDAUS) admixture] with 2,619,936 SNPs and JAP included 66 accessions (28 TRJ, 30 TEJ and 8 *japonica* admixtures) with 1,132,549 SNPs. Aromatics were not included as a panel due to their low abundance. All panels were filtered independently from the full RMC set, excluding alleles with an allele frequency less than 5% and excluding SNPs with 40% percent or greater missing allele calls.

### GWA Parameters for RDP1 and RMC

The software TASSEL version 5.0 (Bradbury et al. 2007) was used to generate a centered identity by state (IBS) kinship matrix and Principal Component Analysis (PCA) for each filtered panel. The number of principal components (PCs) included in the mixed linear model analysis of each set was adjusted as needed to account for population structure within the set. The first three PCs were used as covariates in a mixed linear model for the 395 and “ALL” set of the RDP1 and RMC respectively. For both RDP1 and RMC, 2 PCs were used with the subspecies *Indica* (INDAUS); 1 PC with subspecies *Japonica* (JAP) and IND; and no PCs were used for TEJ, TRJ, and AUS. Mixed linear models were performed in TASSEL version 5.0 (Zhang et al. 2010) with the variance components estimated for each marker and no compression options selected for all panels except the RDP1 395 panel. The RDP1 395 panel was run using the no compression option as well, but P3D was used to estimate heritability for each marker to reduce computation time.

### GWA Mapping Results and Interpretation

A Perl script was used to identify associated chromosome regions from individual SNPs or groups of physically linked SNPs (Huggins et al. 2019). Chromosome regions included 50 Kb in both directions around each individual significant SNP and were extended to include nearby significant SNPs occurring within 200 Kb (Additional file 1: Table S3). The Perl script designated a ‘Peak SNP’ for each region, which corresponded to the SNP with the most significant *p*-value found within the region. An additional Perl script was written to provide the observed frequencies of the alleles present at the peak SNP as well as the allele effect value (Huggins et al. 2019). The R package qqman (Turner, 2014) was used to create Manhattan and quantile–quantile (Q–Q) plots. SNPs with −log10(*p*) ≥ 5 were considered significant and increased stringency was applied during data interpretation as needed, such as when only one SNP in a region met the > −log10(*p*) = 5 threshold, or regions where all associated SNPs had rare alleles (fewer than six accessions).

GWA mapping in multiple panels ascertains chromosomal regions containing QTLs but often a different peak SNP is identified in each QTL region, thus a single “most strongly associated” SNP per QTL is not detected. Rather than report a different candidate gene being detected in each panel, we presumed the simplest solution was usually the best and declared a single QTL/gene in a region and merged the multiple SNPs found associated with one trait from the analysis of multiple panels into a single QTL region for that trait. When merging peaks across panels, a single QTL was declared if the string of SNPs significantly associated for a given trait in one or more of the individual study environments and/or panels did not have a gap greater than ~1.2 Mb and the additive effects were consistent across the SNPs and panels. A chromosomal region containing multiple associated SNPs was declared as two QTL when the gap was more than ~1.2 Mb between associated SNPs, or if the SNP gap was closer than 1.2 Mb but the additive effect changed from positive to negative within a string of associated SNPs (e.g., if the effect of the predominant allele made a switch from positive to negative effect at a defined point within a string of associated SNPs in the same panel). QTL for different traits were considered to overlap if the SNPs significantly associated with the two traits were interspersed with each other or if the ends of the QTL regions were ≤500 Kb apart.

The multiple SNPs identified by GWA analyses for each QTL region were further evaluated using pivot tables in Microsoft Excel to characterize their allele effects in all subpopulation panels as well as on traits in addition to those for which a GWA-QTL was identified. More specifically, for the ShB GWA-QTL, this relationship was examined across the six analyses of the ShB data, AR16-field, AR17-field, ARall-field, NC18-field, AR16-DI and NC18-DI to determine which was the best SNP to target that ShB QTL across the seven panels and studies. Pivot tables were also used to further evaluate if an allele associated by GWA with increased ShB resistance was associated with a desirable or undesirable change in mean TN, PN, days to heading, or height in one or more of the seven panels. When genotypes were used to predict the number of ShB resistance QTL contained by each RDP1 accession, a SNP was considered applicable for a particular accession when the resistance allele was associated with a reduction in mean ShB rating within that accession’s subpopulation panel. In this manner, the SNP utility was extended to more panels than the panel(s) identified by GWA analysis. On occasion, pivot table analysis of various SNPs within a particular GWA-QTL region indicated that a different SNP or allele better predicted the GWA-QTL within different subpopulations.

### Candidate Gene Identification

A Perl script was written to help identify candidate gene(s) within 250 Kb of the significant regions as reported by Huggins et al. (2019), using gene annotations from the Os-Nipponbare-Reference-IRGSP-1.0 assembly (Kawahara et al. 2013; Ouyang et al. 2006), the Rice Annotation Project (RAP1; http://rapdb.dna.affrc.go.jp/, accessed 26 Sept. 2019) (Sakai et al. 2013), and two new annotation files that were created to aid in candidate gene identification. The first file was based on the candidate genes identified in Cohen and Leach (2019), which examined general biotic and abiotic stress response genes in rice. The second file merged all gene annotations from the Os-Nipponbare-Reference-IRGSP-1.0 assembly (Kawahara et al. 2013; Ouyang et al. 2006) (http://rice.uga.edu/pub/data/Eukaryotic_Projects/o_sativa/annotation_dbs/; accessed 26 Sept. 2019) with data in *Oryzabase* (OrzbaseGeneListEn_20190424010057; https://shigen.nig.ac.jp/rice/oryzabase/download/gene; accessed 26 Sept. 2019) that shared a unique RAP ID. Also, an additional Perl script was written to examine significant chromosome regions that overlapped among the different traits and panels (Huggins et al. 2019).

## Supplementary Information

**Additional file 1. Table S1:** Percentages of the entire RDP1, and of each subpopulation that were classified as resistant (R), moderately resistant (MR), moderately susceptible (MS), and susceptible (S) based on their field and microchamber disease index (DI) sheath blight (ShB) severity scores. Percentages based on height, heading, tillering, and panicle number are also shown. *O. sativa* subpopulations are: *aus* (AUS), *indica* (IND), *temperate japonica* (TEJ), *tropical japonica* (TRJ), aromatic (ARO). **Table S2:** Pearson correlations across all RDP1 (same as Table 2) and subpopulation panels. Correlation values (r) are blue if significantly positive, red if significantly negative, and black if not significant at α = 0.05. Significance at α = 0.05, α = 0.01, and α = 0.001 are indicated with *, **, and ***, respectively. Sheath blight (ShB) response was evaluated in Arkansas, USA (AR) and Nanning, China (NC) using both field scoring and microchamber disease index (DI). Plant height and days to heading were evaluated in the same AR and NC field plots. Culm habit was rated only in AR. Tiller and panicle number per plant were evaluated in potted plants grown in the greenhouse. Tiller number was counted in young plants at the 5- to 6-week age (mid-tillering stage), panicle number was determined from the same plants grown to maturity. **Table S3:** List of all the trait associated SNPs identified by the GWA analysis in chromosomal order. The 18 sheath blight (ShB) QTL regions, 15 tiller number (TN) QTL and 14 panicle number (PN) QTL reported in this study are identified in the first three columns, respectively. **Table S4:** List of the trait-associated SNPs in the QTL regions identified by GWA for ShB (sheath blight), TN (tiller number), and PN (panicle number) selected from the SNPs listed in Table S3. In the QTL column, the yellow highlight identifies the targeted peak SNP within the QTL region reported in Table 3 (ShB-QTL) or Table 4 (TN-QTL, PN-QTL). The starting and ending SNP are in bold to indicate the beginning and end of the QTL region. **Table S5:** Comparison of the targeted SNP reference allele frequency in the selected RDP1 panel to the reference allele frequencies across the five rice subpopulations for the 4,726 *O. sativa* accessions included in the RiceVarMap v2.0 database (Zhao et al. 2014). **Table S6:** A summary of quantitative trait loci (QTL) for sheath blight resistance reported in other GWA and biparental studies that were in the same regions as the ShB GWA-QTL reported in this study. [QTL identified in biparental populations are based on reviews by Jia et al. (2009), Molla et al. (2020) and Srinivasachary et al. (2011).]. **Table S7:** List of all the significant peak SNPs for ShB identified by the GWA analyses of the RDP1 (present data) and RMC using the ShB-DI LSmeans reported in Jia et al. (2012) and the genotypes for 167 RMC accessions based on 3,200,320 SNPs. The RDP1 GWA-QTL and previously reported SSR markers significantly associated with ShB (Jia et al. 2012) are listed in the first column. The "RMC SNP GWA QTL" column identifies the GWA-QTL based on the SNP genotypes for 167 RMC accessions as reported in this study, and the starting and ending SNPs of the QTL region are highlighted in dark magenta. (RMC is the Rice Minicore association mapping panel). **Table S8:** Phenotypic and genotypic data for the Rice Diversity Panel 1 (RDP1) accessions used for GWA-mapping that identified 18 QTL for rice sheath blight (ShB) resistance. Phenotypes provided are LSmeans calculated across replications for ShB-scores collected in Arkansas, USA (AR) and Nanning, China (NC) in field plots and using a microchamber disease index (DI) method. Plant height and days to heading (DH) were measured in ShB inoculated field plots. Tiller number (TN) BLUEs were calculated across three replications of greenhouse grown plants 5- to 6-weeks of age at time of tiller counting. Also provided are the Genetics Stocks-*Oryza* (GSOR) accession numbers and names, originating country, and subpopulation group based on Wang et al. (2018). Three sets of example selections are provided, one based on a combination of AR-field and NC-field performance, a second based on AR-DI and NC-DI scores, and a third based on the number of R alleles estimated in each accession based on QTL-related SNP alleles (Table S4). For estimating the total number of R alleles contained in each RDP1 at the 18 reported ShB-QTL, accessions were estimated to contain the R allele for a QTL when it contained an R-associated allele for one or more peak SNP(s) per QTL, as detailed in columns AM to BG per subpopulation. 'Fail' indicates a failed reaction thus, no SNP genotype was called for a particular accession, 'na' indicates a particular QTL was not applicable to accessions in that subpopulation group. **Table S9:** Phenotypic gains from selections based on marker-predicted number of R-alleles for each accession and summarized by subpopulation and number of R-alleles. Phenotypes provided are LSmeans calculated across replications for ShB scores collected in Arkansas, USA (AR) and Nanning, China (NC) in field plots and using a microchamber disease index (DI) method. Plant height and days to heading (DH) were measured in ShB inoculated field plots. Tiller number data are BLUEs calculated across three replications of plants whose tillers were counted at 5- to 6-weeks of age, the mid-tillering stage

**Additional file 2. Fig. S1:** Genome-wide association (GWA) mapping results for rice sheath blight disease ratings based on an imputed 3,463,224 SNP dataset. Field and greenhouse studies were conducted in both Stuttgart, Arkansas (AR) USA and Nanning, China (NC) with Arkansas field studies being conducted in 2016 and 2017, and Nanning field studies in 2018. Manhattan (left) and Q-Q (right) plots are grouped by study environment a) Arkansas field 2016, b) Arkansas field 2017, c) Arkansas field both years combined, d) Nanning field 2018, e) Arkansas greenhouse, and f) Nanning greenhouse. Within each environment the plots are arranged in the following order by panel *Indica* subspecies (INDAUS), *aus* (AUS), *Indica* (IND), *Japonica* subspecies (JAP), *temperate japonica* (TEJ), *tropical japonica* (TRJ) and all RDP1 accessions (395). In the Manhattan plots the X axis shows the SNP positions across the 12 rice chromosomes and the Y axis is the –log10 (*p*) value for each SNP. The black horizontal line represents the –log10(*p*) significance threshold at 5. Black arrows identify the significant peak SNPs with regions >100 Kb comprising the 18 GWA ShB-QTL. The 21 target SNPs listed in Table 3 are identified by red arrows. (If two or more peak SNPs were in close proximity in a given QTL region, the peaks were denoted by a single arrow.) **Fig. S2:** Genome-wide association (GWA) mapping results for days to 50% heading, plant height and culm habit based on an imputed 3,463,224 SNP dataset. The data was collected from field studies conducted at Stuttgart, Arkansas (AR) in 2016 and 2017, and Nanning, China (NC) in 2018. Manhattan (left) and Q-Q (right) plots are grouped by the trait evaluated in the field study as follows: a) days to heading, b) plant height and c) culm habit. Within each trait the plots are arranged by field study environment Arkansas field 2016 (AR16), Arkansas field 2017 (AR17), Arkansas field both years (ARall) and Nanning field 2018 (NC18), Culm habit was only recorded from the Arkansas field study in 2017. In the Manhattan plots the X axis shows the SNP positions across the 12 rice chromosomes and the Y axis is the –log10 (*p*) value for each SNP. The black horizontal line represents the –log10(*p*) significance threshold at 5. Black arrows indicate the significant peak SNPs identified by the GWA mapping of the RDP1 accessions (395) with a region >100 Kb. Details are in Table S3. (If two or more peak SNPs were in close proximity, they were denoted by a single arrow.) **Fig. S3:** Manhattan (left) and Q-Q (right) plots identifying the 15 TN and 14 PN QTL regions identified by GWA analyses using an imputed 3,463,244 SNP dataset (Table 4). In the Manhattan plots the X axis shows the SNP positions across the 12 rice chromosomes and the Y axis is the –log10 (*p*) value for each SNP. The black horizontal line represents the –log10(*p*) significance threshold at 5. Black arrows indicate peak SNPs within the indicated QTL regions. The plots are organized by trait with the TN results in plots A through F and PN results in plots in G through L. Subpopulation abbreviations are INDAUS (*Indica* subspecies), JAP (*Japonica* subspecies), AUS (*aus*), IND (*Indica*), TEJ (*temperate japonica*), and TRJ (*tropical japonica*)

## Data Availability

The data is included in the Additional files 1 and 2 associated with this manuscript. The Rice Diversity Panel 1 and the Rice Minicore collection are available from the USDA-ARS Genetic Stocks-*Oryza* (GSOR) collection located at the USDA-ARS Dale Bumpers National Rice Research Center (DBNRRC), Stuttgart, Arkansas USA (www.ars.usda.gov/GSOR). Presidio (PI636465), the low tillering check in the tillering studies, is available from the USDA-ARS National Small Grains Collection and requested through GRIN-Global (https://ars-grin.gov/npgs).

## Abbreviations

3K RGP: 3,000 Rice Genomes Project
Admix: Admixture
AR: Arkansas, USA
ARO: Aromatic subpopulation
AUS: *aus* subpopulation
BLUE: Best linear unbiased estimate
BLUP: Best linear unbiased prediction
DI: Disease index
gh: Greenhouse
GWA: Genome-wide association
IND: *indica* subpopulation
INDAUS: *Indica* subspecies
JAP: *Japonica* subspecies
LSmean: Least squares mean
NC: Nanning, China
PN: Panicle number
QTL: Quantitative trait locus
R: Resistant
RDP1: Rice diversity panel 1
RMC: Rice Minicore
ShB: Sheath blight
S: Susceptible
SNP: Single nucleotide polymorphism
SSR: Simple sequence repeat
TEJ: *temperate japonica* subpopulation
TN: Tiller number
TRJ: *tropical japonica* subpopulation
