# Supplementary material for: Assessment of Rice Sheath Blight Resistance Including Associations with Plant Architecture, as Revealed by Genome-Wide Association Studies
Source: Rice (N Y). 2022 Jun 18;15:31. doi: 10.1186/s12284-022-00574-4 (PMC9206596; doi:10.1186/s12284-022-00574-4)

Supplementary Figures S1, S2 and S3 for:

Assessment of Rice Sheath Blight Resistance including Associations with Plant Architecture, as Revealed by Genome-wide Association Studies

Danting Li, Fantao Zhang, Shannon R.M. Pinson, Jeremy D. Edwards, Aaron K. Jackson, Xiuzhong Xia and Georgia C. Eizenga

Fig. S1: Genome-wide association (GWA) mapping results for rice sheath blight disease ratings based on an imputed 3,463,224 SNP dataset. Field and greenhouse studies were conducted in both Stuttgart, Arkansas (AR) USA and Nanning, China (NC) with Arkansas field studies being conducted in 2016 and 2017, and Nanning field studies in 2018. Manhattan (left) and Q-Q (right) plots are grouped by study environment a) Arkansas field 2016, b) Arkansas field 2017, c) Arkansas field both years combined, d) Nanning field 2018, e) Arkansas greenhouse, and f) Nanning greenhouse. Within each environment the plots are arranged in the following order by panel *Indica* subspecies (INDAUS), *aus* (AUS), *indica* (IND), *Japonica* subspecies (JAP), *temperate japonica* (TEJ), *tropical japonica* (TRJ) and all RDP1 accessions (395). In the Manhattan plots the X axis shows the SNP positions across the 12 rice chromosomes and the Y axis is the –log10 (p) value for each SNP. The black horizontal line represents the –log10(p) significance threshold at 5. Black arrows identify the significant peak SNPs with regions >100 Kb comprising the 18 GWA ShB-QTL. The 21 target SNPs listed in Table 3 are identified by red arrows. (If two or more peak SNPs were in close proximity in a given QTL region, the peaks were denoted by a single arrow.)

Fig. S1a: Field evaluation conducted in 2016 at Stuttgart, Arkansas USA

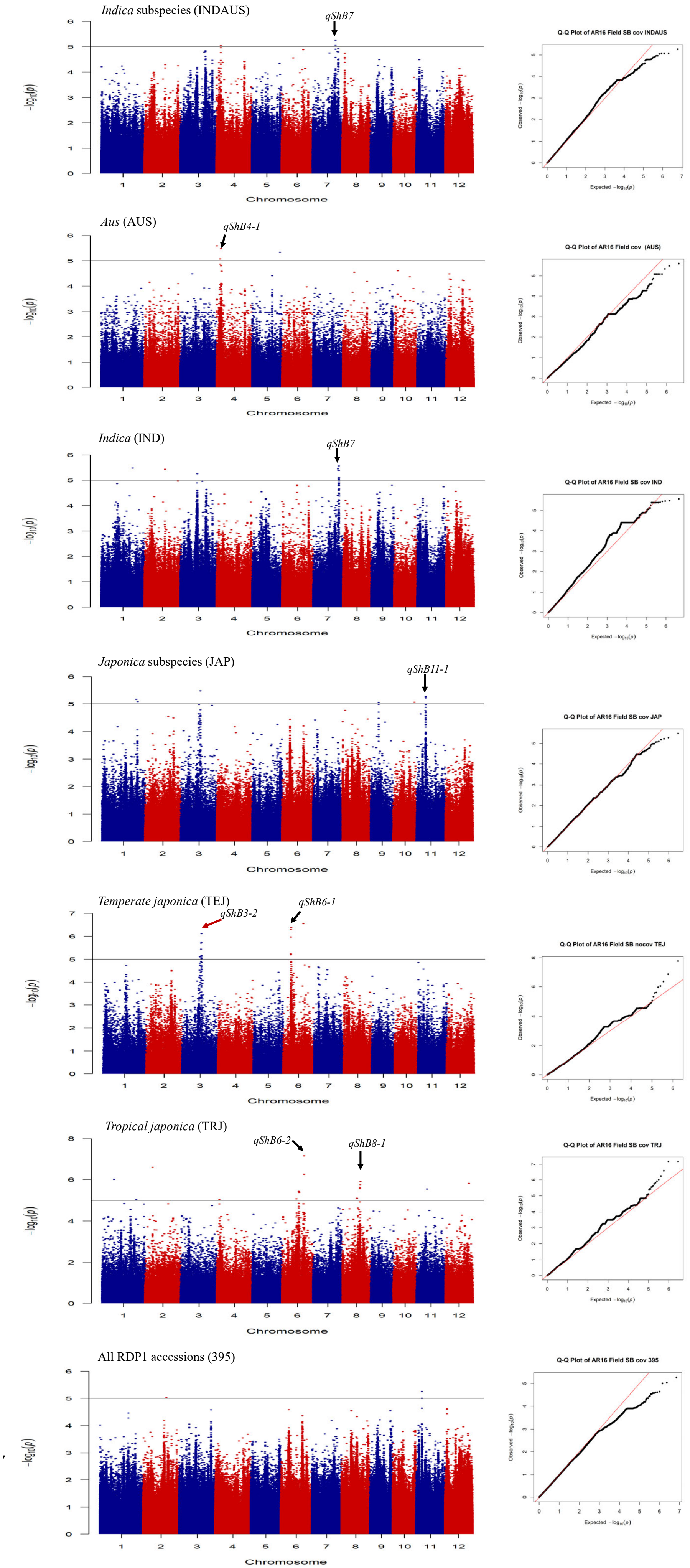

Fig. S1b: Field evaluation conducted in 2017 at Stuttgart, Arkansas USA

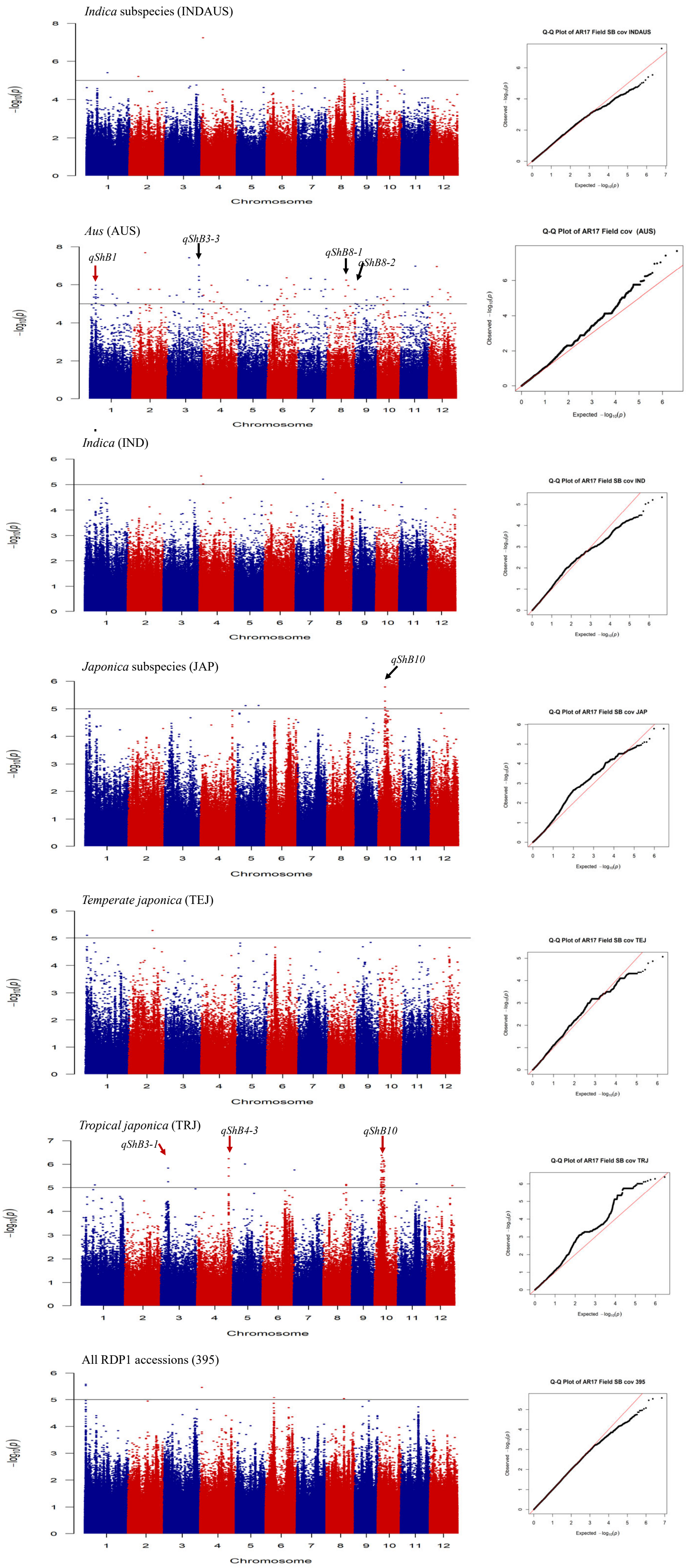

Fig. S1c: Combined field evaluations conducted in 2016 and 2017 at Stuttgart, Arkansas USA

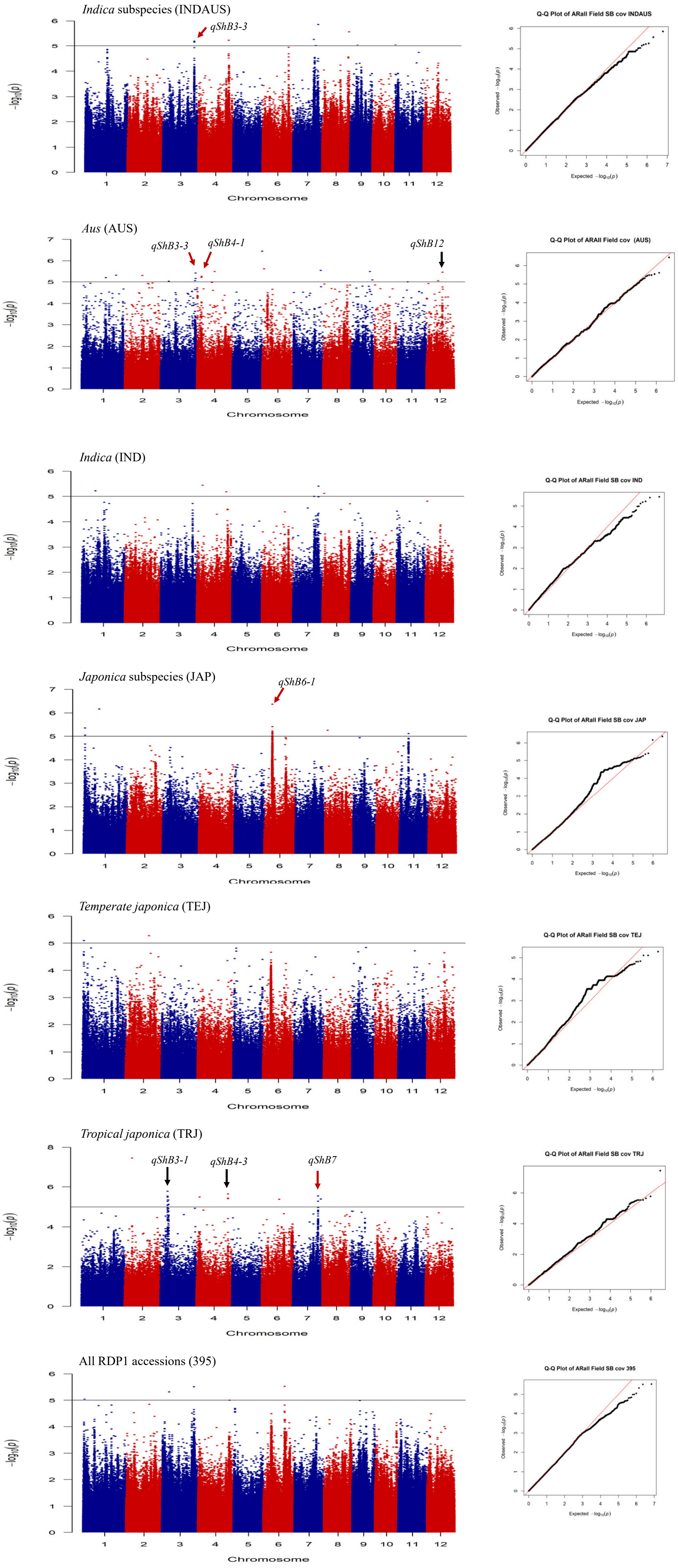

Fig. S1d: Field evaluation conducted in 2018 at Nanning, China

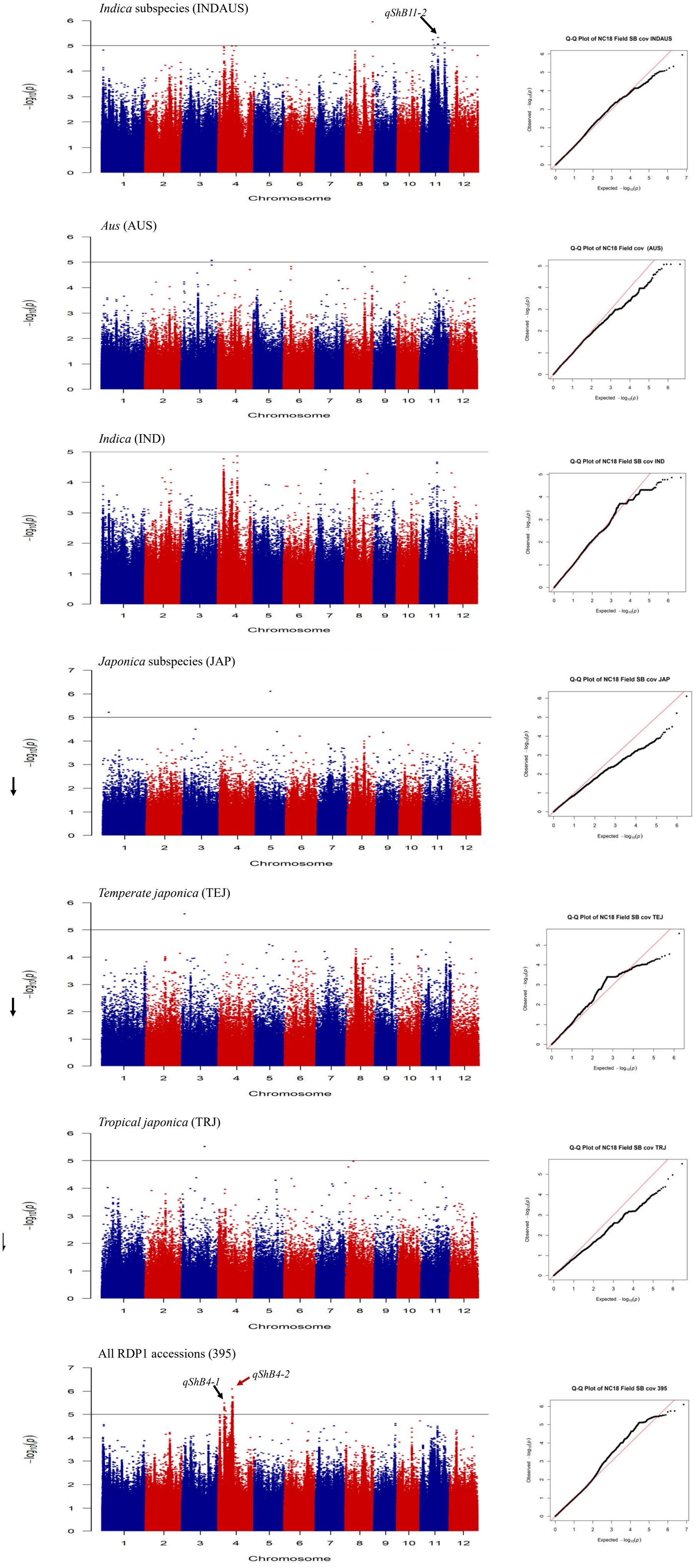

Fig. S1e: Greenhouse evaluation conducted at Stuttgart. Arkansas USA using the microchamber method

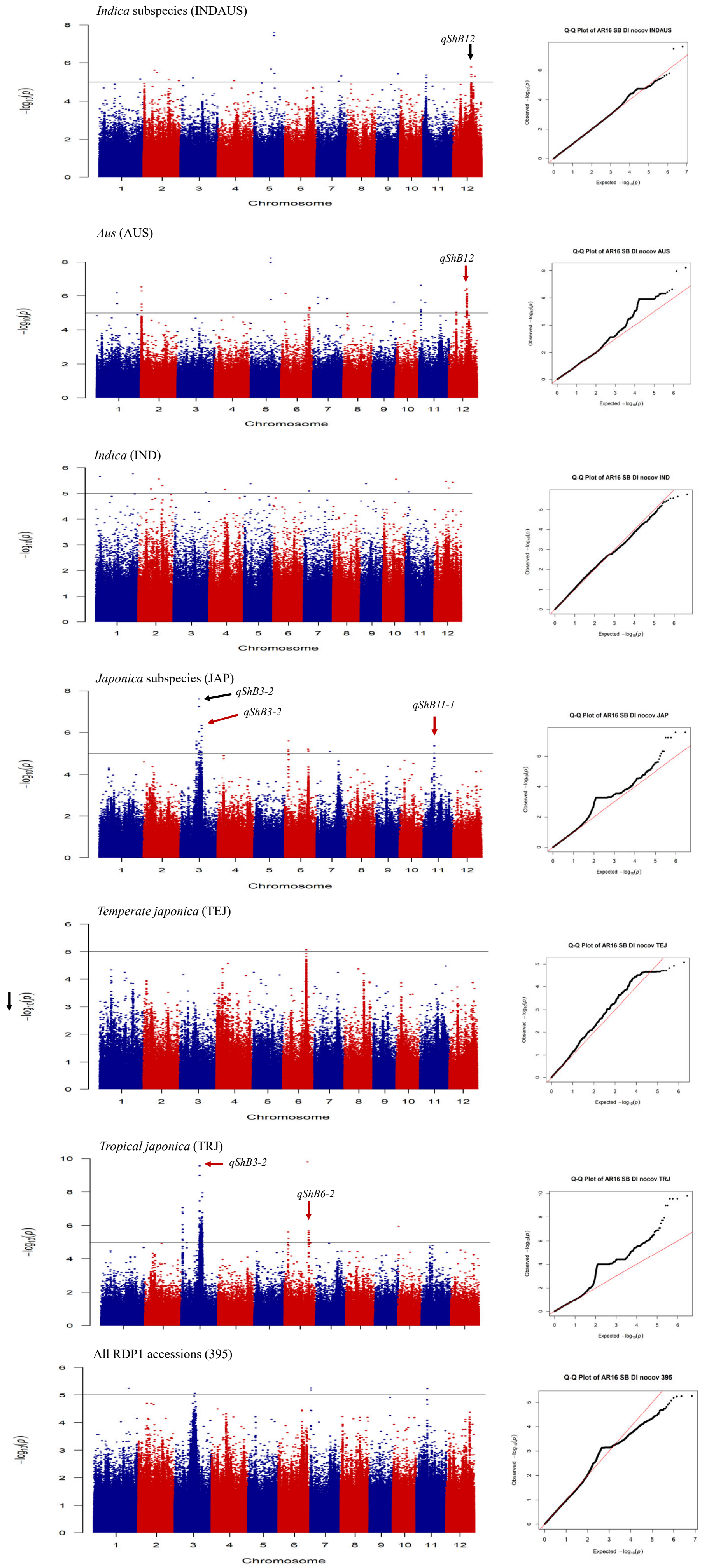

Fig. S1f: Greenhouse evaluation conducted at Nanning, China using the microchamber method

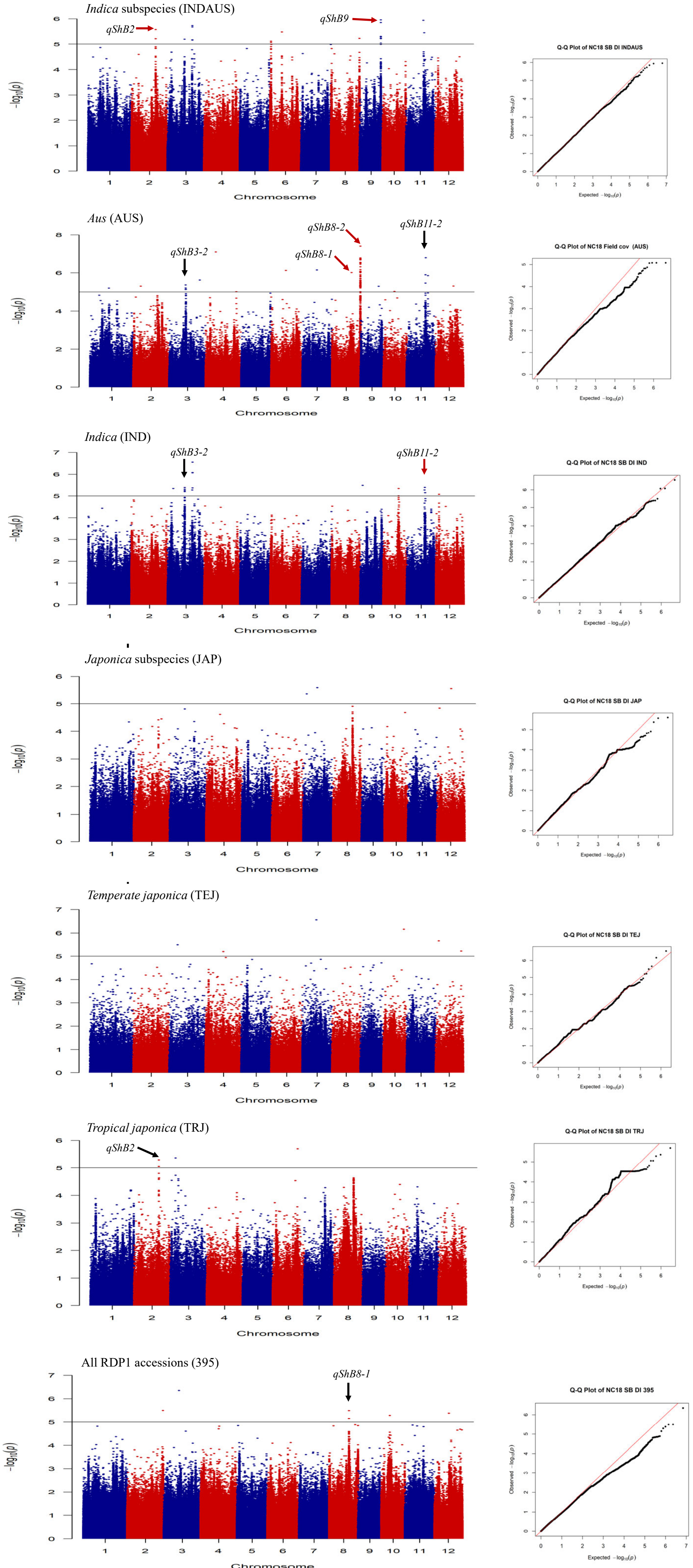

Fig. S2: Genome-wide association (GWA) mapping results for days to 50% heading, plant height and culm habit based on an imputed 3,463,224 SNP dataset. The data was collected from field studies conducted at Stuttgart, Arkansas (AR) in 2016 and 2017, and Nanning, China (NC) in 2018. Manhattan (left) and Q-Q (right) plots are grouped by the trait evaluated in the field study as follows: a) days to heading, b) plant height and c) culm habit. Within each trait the plots are arranged by field study environment Arkansas field 2016 (AR16), Arkansas field 2017 (AR17), Arkansas field both years (ARall) and Nanning field 2018 (NC18). Culm habit was only recorded from the Arkansas field study in 2017. In the Manhattan plots the X axis shows the SNP positions across the 12 rice chromosomes and the Y axis is the –log10 (*p*) value for each SNP. The black horizontal line represents the –log10(*p*) significance threshold at 5. Black arrows indicate the significant peak SNPs identified by the GWA mapping of the RDP1 accessions (395) with a region >100 Kb. Details are in Table S3. (If two or more peak SNPs were in close proximity, they were denoted by a single arrow.)

Fig. S2a: Days to 50% heading measured in field studies conducted in Arkansas in 2016 (AR16), Arkansas in 2017 (AR17), Arkansas both in 2016 and 2017, and Nanning in 2018 (NC18). Black arrows are the significant merged SNP peaks

Measured during the 2016 field season near Stuttgart, Arkansas, USA

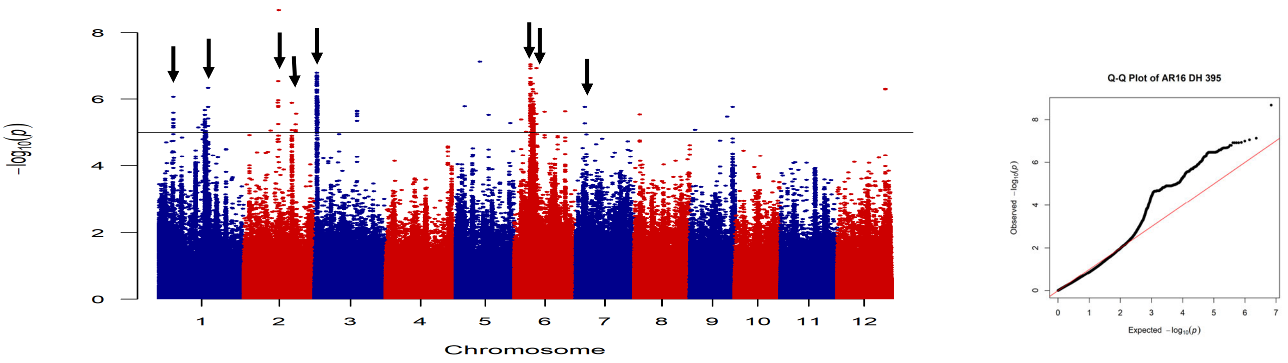

Measured during the 2017 field season near Stuttgart, Arkansas, USA

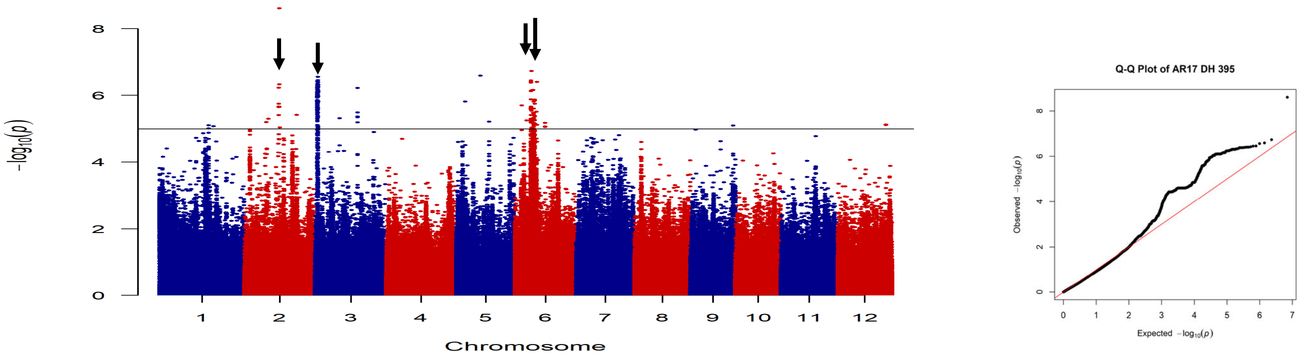

Combined mean of the measurements during the 2016 and 2017 field seasons near Stuttgart, Arkansas, USA

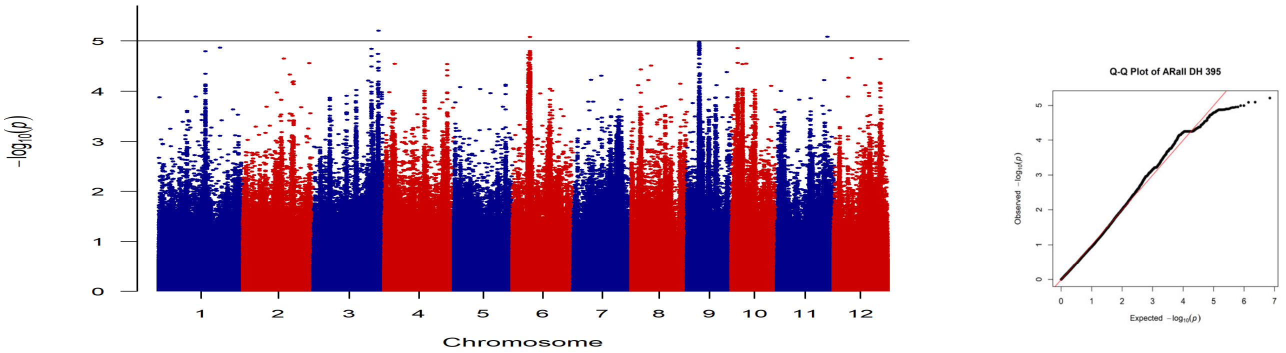

Measured during the 2018 field season near Nanning, China

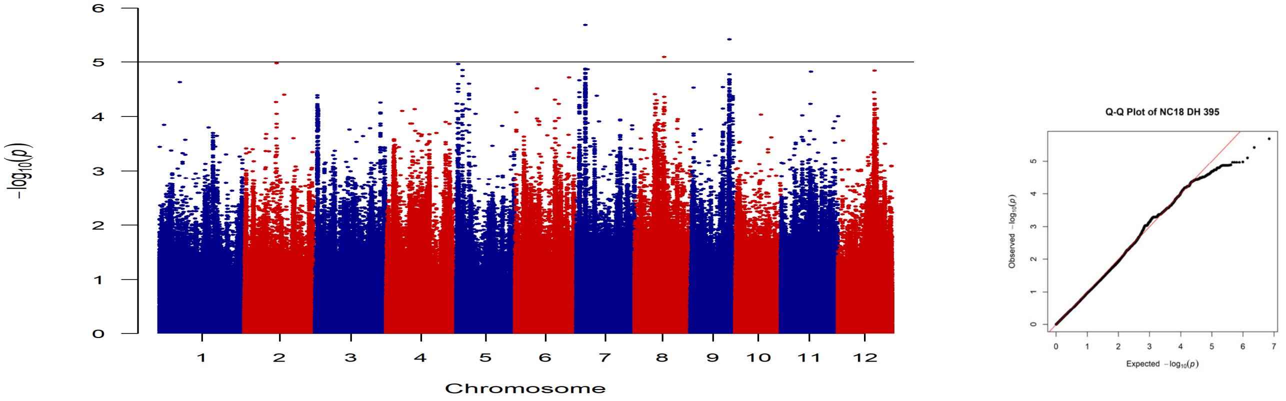

Fig. S2b: Plant height measured in field studies conducted in Arkansas in 2016 (AR16), Arkansas in 2017 (AR17), Arkansas both in 2016 and 2017, and Nanning in 2018 (NC18). Black arrows are the significant merged SNP peaks

Measured during the 2016 field season near Stuttgart, Arkansas, USA

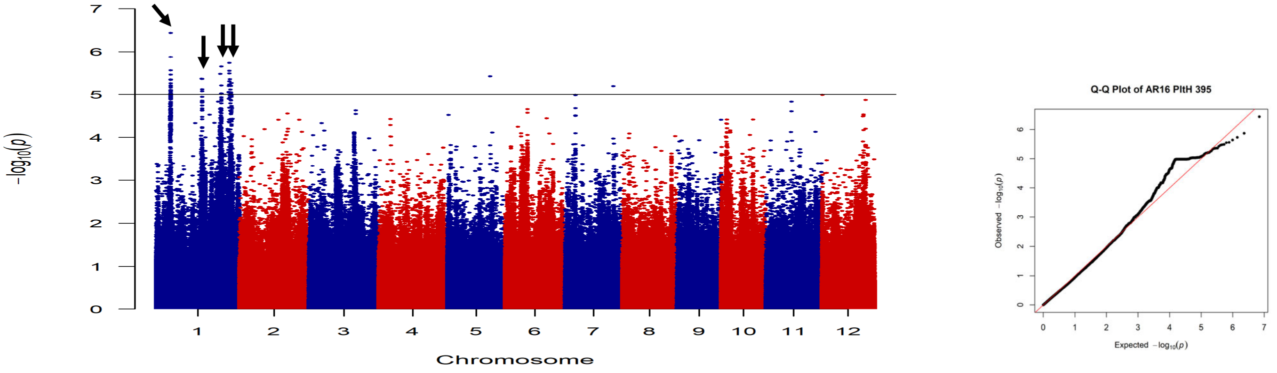

Measured during the 2017 field season near Stuttgart, Arkansas, USA

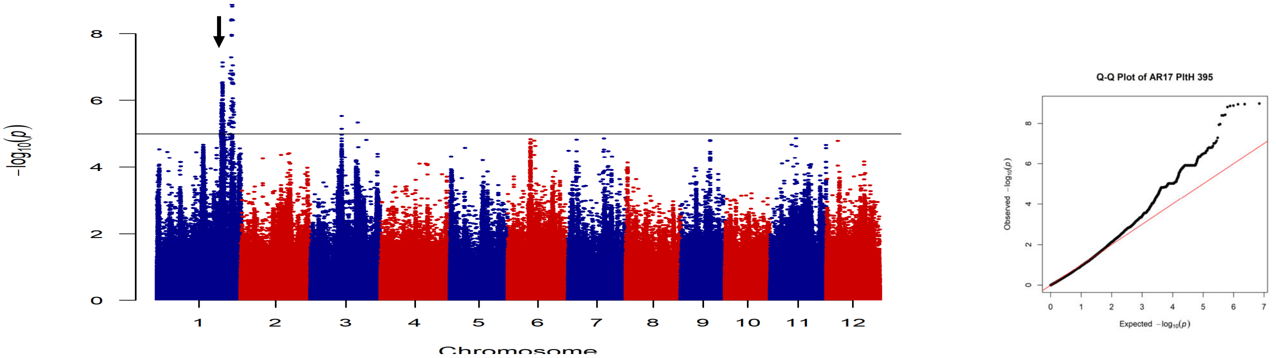

Combined mean of the measurements during the 2016 and 2017 field seasons near Stuttgart, Arkansas, USA

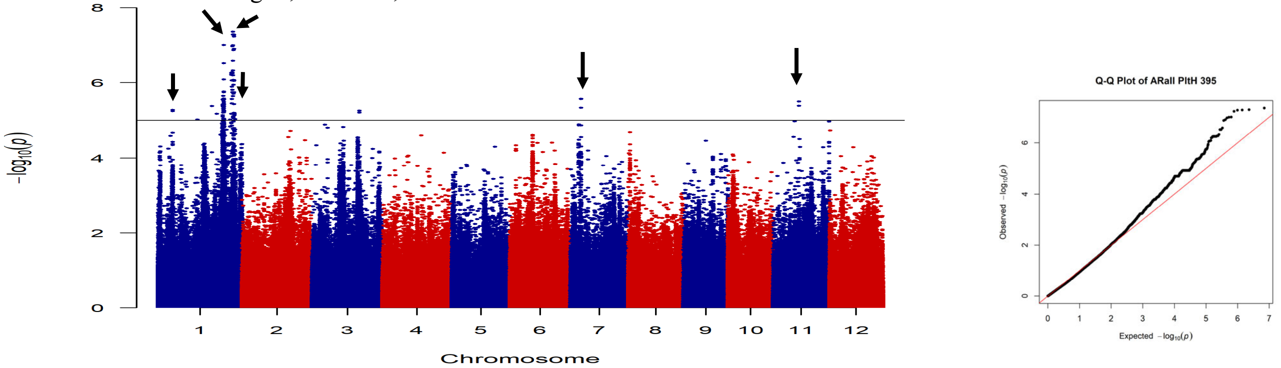

Measured during the 2018 field season near Nanning, China

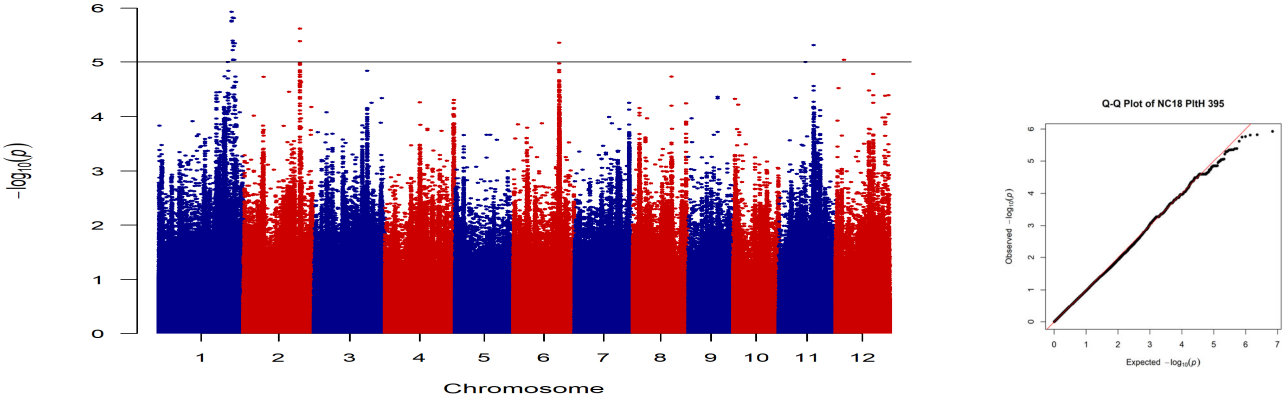

Fig. S2c: Culm habit only rated in the field study conducted at Stuttgart, Arkansas in 2017 (AR17). Black arrow is the only significant SNP identified

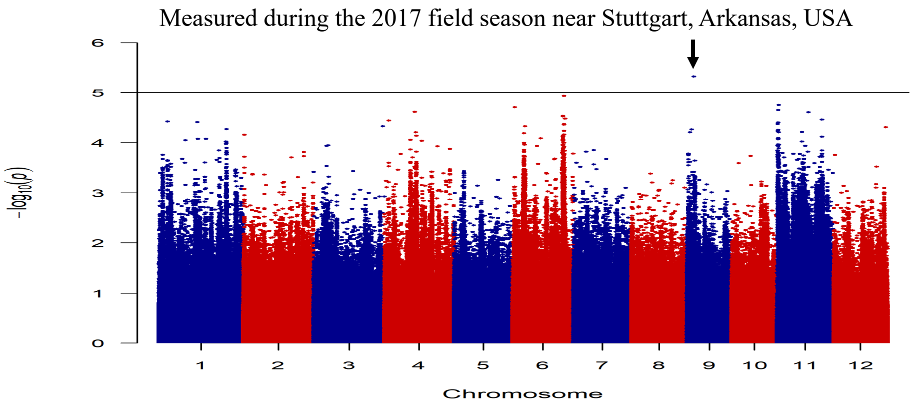

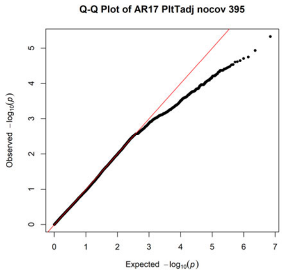

Fig. S3: Manhattan (left) and Q-Q (right) plots identifying the 15 TN and 14 PN QTL regions identified by GWA analyses using an imputed 3,463,244 SNP dataset (Table 4). In the Manhattan plots the X axis shows the SNP positions across the 12 rice chromosomes and the Y axis is the –log10 (p) value for each SNP. The black horizontal line represents the –log10(p) threshold at 5. Black arrows indicate peak SNPs within the indicated QTL regions. The plots are organized by trait with the TN results in plots A through F and PN results in plots in G through L. Subpopulation abbreviations are INDAUS (*Indica* subspecies), JAP (*Japonica* subspecies), AUS (*aus*), IND (*indica*), TEJ (*temperate japonica*), and TRJ (*tropical japonica*)

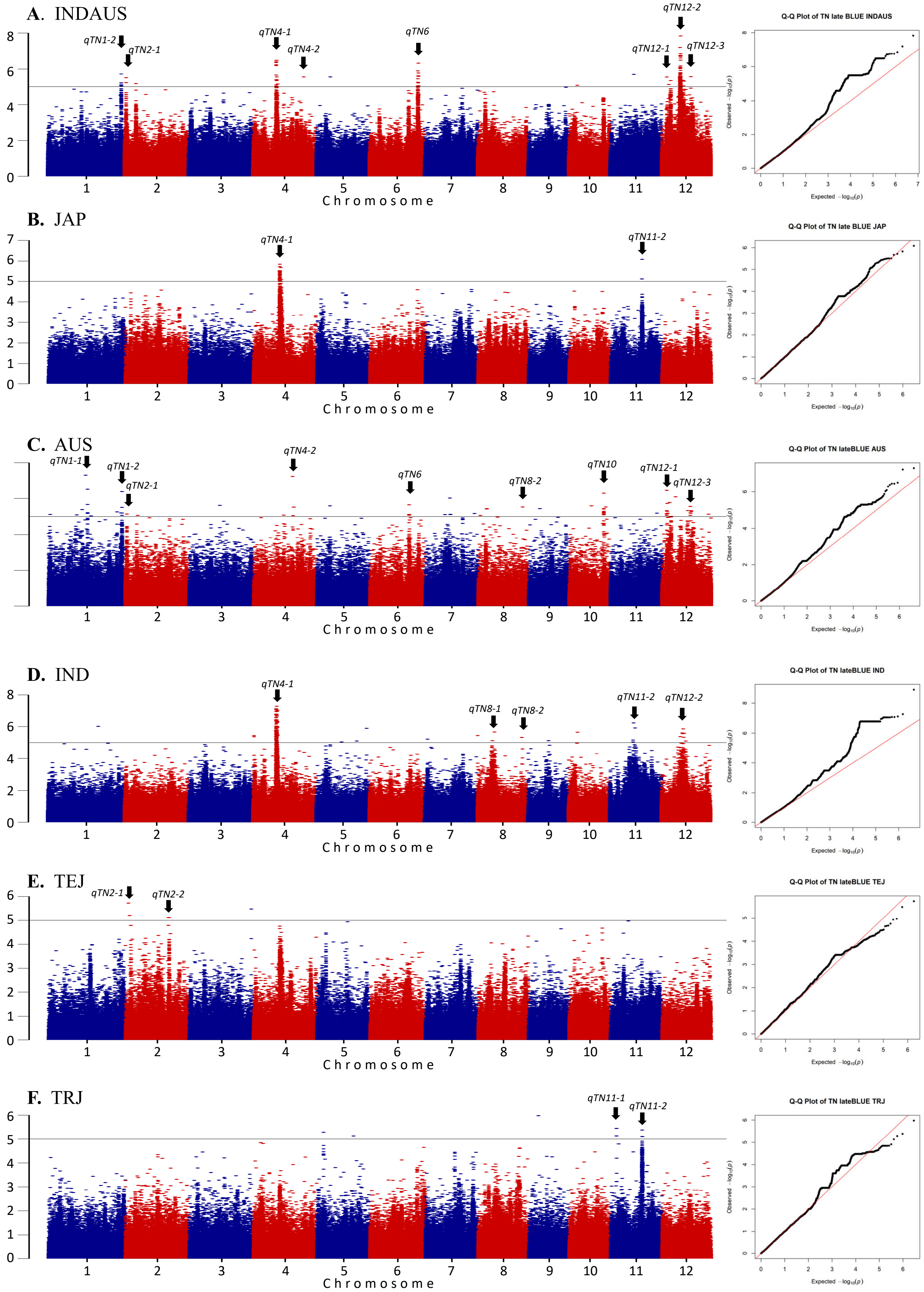

Fig. S3 continued. Manhattan (left) and Q-Q (right) plots identifying the 15 TN and 14 PN QTL regions identified by GWA analyses (Table 5).

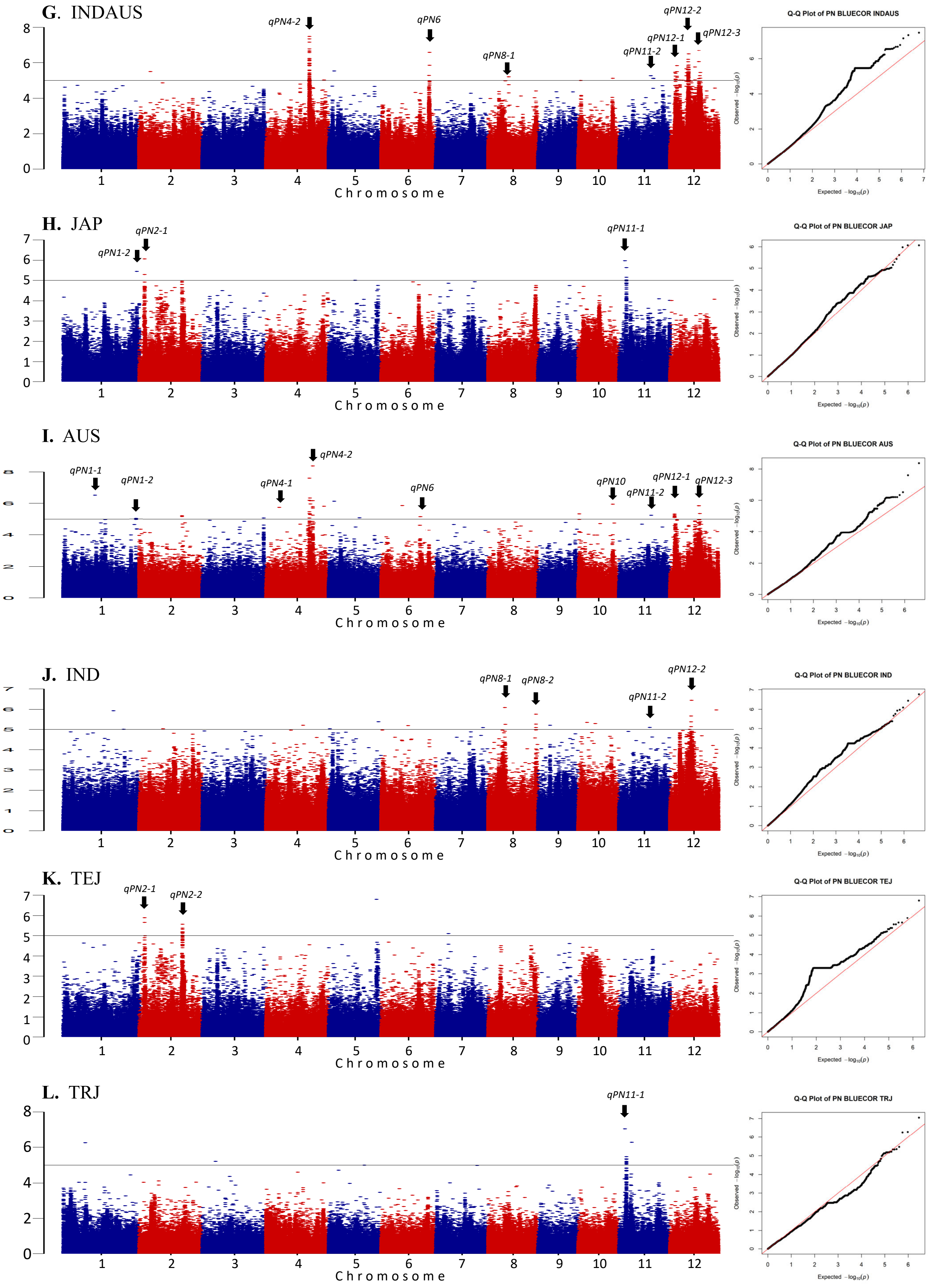

Supplement: Supplementary file 2 — Additional file 2. Fig. S1: Genome-wide association (GWA) mapping results for rice sheath blight disease ratings based on an imputed 3,463,224 SNP dataset. Field and greenhouse studies were conducted in both Stuttgart, Arkansas (AR) USA and Nanning, China (NC) with Arkansas field studies being conducted in 2016 and 2017, and Nanning field studies in 2018. Manhattan (left) and Q-Q (right) plots are grouped by study environment a) Arkansas field 2016, b) Arkansas field 2017, c) Arkansas field both years combined, d) Nanning field 2018, e) Arkansas greenhouse, and f) Nanning greenhouse. Within each environment the plots are arranged in the following order by panel Indica subspecies (INDAUS), aus (AUS), Indica (IND), Japonica subspecies (JAP), temperate japonica (TEJ), tropical japonica (TRJ) and all RDP1 accessions (395). In the Manhattan plots the X axis shows the SNP positions across the 12 rice chromosomes and the Y axis is the –log10 (p) value for each SNP. The black horizontal line represents the –log10(p) significance threshold at 5. Black arrows identify the significant peak SNPs with regions >100 Kb comprising the 18 GWA ShB-QTL. The 21 target SNPs listed in Table 3 are identified by red arrows. (If two or more peak SNPs were in close proximity in a given QTL region, the peaks were denoted by a single arrow.) Fig. S2: Genome-wide association (GWA) mapping results for days to 50% heading, plant height and culm habit based on an imputed 3,463,224 SNP dataset. The data was collected from field studies conducted at Stuttgart, Arkansas (AR) in 2016 and 2017, and Nanning, China (NC) in 2018. Manhattan (left) and Q-Q (right) plots are grouped by the trait evaluated in the field study as follows: a) days to heading, b) plant height and c) culm habit. Within each trait the plots are arranged by field study environment Arkansas field 2016 (AR16), Arkansas field 2017 (AR17), Arkansas field both years (ARall) and Nanning field 2018 (NC18), Culm habit was only recorded from [file 12284_2022_574_MOESM2_ESM.pdf]
